# Quantitative MRI maps of human neocortex explored using cell type-specific gene expression analysis

**DOI:** 10.1093/cercor/bhac453

**Published:** 2022-12-15

**Authors:** Luke J Edwards, Peter McColgan, Saskia Helbling, Angeliki Zarkali, Lenka Vaculčiaková, Kerrin J Pine, Fred Dick, Nikolaus Weiskopf

**Affiliations:** Department of Neurophysics, Max Planck Institute for Human Cognitive and Brain Sciences, Leipzig, DE, Germany; Department of Neurophysics, Max Planck Institute for Human Cognitive and Brain Sciences, Leipzig, DE, Germany; Huntington’s Disease Centre, University College London, London, UK; Department of Neurophysics, Max Planck Institute for Human Cognitive and Brain Sciences, Leipzig, DE, Germany; Poeppel Lab, Ernst Strüngmann Institute (ESI) for Neuroscience in Cooperation with Max Planck Society, Frankfurt am Main, DE, Germany; Dementia Research Centre, University College London, London, UK; Department of Neurophysics, Max Planck Institute for Human Cognitive and Brain Sciences, Leipzig, DE, Germany; Department of Neurophysics, Max Planck Institute for Human Cognitive and Brain Sciences, Leipzig, DE, Germany; Birkbeck/UCL Centre for Neuroimaging (BUCNI), London, UK; Department of Neurophysics, Max Planck Institute for Human Cognitive and Brain Sciences, Leipzig, DE, Germany; Felix Bloch Institute for Solid State Physics, Faculty of Physics and Earth Sciences, Leipzig University, Leipzig, DE, Germany

**Keywords:** hMRI, isocortex, magnetic resonance imaging, myelin, relaxometry

## Abstract

Quantitative magnetic resonance imaging (qMRI) allows extraction of reproducible and robust parameter maps. However, the connection to underlying biological substrates remains murky, especially in the complex, densely packed cortex. We investigated associations in human neocortex between qMRI parameters and neocortical cell types by comparing the spatial distribution of the qMRI parameters longitudinal relaxation rate (${R_{1}}$), effective transverse relaxation rate (${R_{2}}^{\ast }$), and magnetization transfer saturation (MTsat) to gene expression from the Allen Human Brain Atlas, then combining this with lists of genes enriched in specific cell types found in the human brain. As qMRI parameters are magnetic field strength-dependent, the analysis was performed on MRI data at 3T and 7T. All qMRI parameters significantly covaried with genes enriched in GABA- and glutamatergic neurons, i.e. they were associated with cytoarchitecture. The qMRI parameters also significantly covaried with the distribution of genes enriched in astrocytes (${R_{2}}^{\ast }$ at 3T, ${R_{1}}$ at 7T), endothelial cells (${R_{1}}$ and MTsat at 3T), microglia (${R_{1}}$ and MTsat at 3T, ${R_{1}}$ at 7T), and oligodendrocytes and oligodendrocyte precursor cells (${R_{1}}$ at 7T). These results advance the potential use of qMRI parameters as biomarkers for specific cell types.

## Introduction

Multiparameter mapping (MPM) protocols allow rapid and efficient acquisition of relaxometric quantitative magnetic resonance imaging (qMRI) parameters in vivo (Tabelow et al. 2019) robustly and reproducibly (Leutritz et al. 2020). These parameters include the longitudinal relaxation rate (${R_{1}}$), magnetization transfer saturation (MTsat), and effective transverse relaxation rate (${R_{2}}^{\ast }$). In vivo histology aims to take such quantitative maps and extract information about the underlying microscopic biological substructures beyond the resolution of MRI (Edwards et al. 2018, Weiskopf et al. 2021).

On the whole-brain level, contrast in the above qMRI parameters is mainly driven by two main sources: macromolecules (mostly myelin), and iron (these sources of contrast are reviewed in Edwards et al. (2018), Möller et al. (2019), and Weiskopf et al. (2021)). MTsat is interpreted as a marker for macromolecules (Georgiadis et al. 2021), ${R_{2}}^{\ast }$ is interpreted as a marker for mainly iron content with some sensitivity to macromolecules (Fukunaga et al. 2010, Kirilina et al. 2020, Langkammer et al. 2010), and ${R_{1}}$ is interpreted as a marker for mainly macromolecular content with some sensitivity to iron (Callaghan et al. 2015, Stüber et al. 2014). The sensitivity of qMRI parameters to different sources is known to vary with the static magnetic field strength (Peters et al. 2007, Rooney et al. 2007, Wang et al. 2020).

These relatively simple interpretations of qMRI parameters do not, however, allow us to infer information about the cellular architecture of the brain from their values. On the cellular level, the human neocortex is a complex, densely packed structure containing billions of neurons and glia (Lent et al. 2012). The distribution of these neurons and glia varies over the brain, forming laminae and cortical areas that can be distinguished under the microscope (reviewed from an MRI perspective in Edwards et al. (2018)). Herein we aim to investigate the relationship between neocortical cellular architecture and qMRI parameters by comparing spatial differences in expression of cell type-specific genes with the spatial distribution of the qMRI parameters ${R_{1}}$, MTsat, and ${R_{2}}^{\ast }$.

Gene expression differences reflect and determine different cell types; differential gene expression throughout the neocortex can thus be related to differential expression of cell types (Arnatkevi˘ciūtė et al. 2019, Fornito et al. 2019, Lein et al. 2017). The combination of knowledge of cell type-specific genes (Hawrylycz et al. 2015, Hodge et al. 2019, Zeisel et al. 2015) with the cortical gene expression results found in the Allen Human Brain Atlas (AHBA) of gene expression (Hawrylycz et al. 2012) from the Allen Institute for Brain Science (AIBS) has shed light on the biological substrates of several different MRI parameters in the cortex, e.g. Liu et al. (2019), McColgan et al. (2021), Patel et al.(2019, 2020), Shin et al. (2018), Wen et al. (2018), Whitaker et al. (2016).

Of these prior works, those which are most relevant for this study are Patel et al. (2020) and Wen et al. (2018), which both used relationships between gene expression and cellular architecture to shed light on cell-type substrates of quantitative MRI metrics. We briefly summarise these works here in order to lay the groundwork for the rest of the manuscript and allow us to differentiate our approach from previous approaches.

Patel et al. (2020) examined the cell type-specific associations with a number of different qMRI parameters at a static magnetic field strength of 3T, including ${R_{1}}$ and a measure of magnetization transfer (the magnetization transfer ratio, MTR) in a large young male cohort. Despite the common biophysical interpretation of these two parameters as markers of macromolecular content, and thus predominantly of myelin, no significant association was found to the oligodendrocyte cells (ODCs) that build myelin (Möller et al. 2019). Instead, ${R_{1}}$ was associated with gene expression enriched in astrocytes and CA1-pyramidal neurons, and MTR was associated with gene expression enriched in CA1-pyramidal neurons and S1-pyramidal neurons (Patel et al. 2019). This accords with our observations using an MPM protocol at 3T in a smaller healthy adult cohort using a similar method of gene expression analysis (Edwards et al. 2019). Interestingly, Patel et al. (2020) found that maps of transverse relaxation rate (${R_{2}}$) and the ${R_{2}}$-derived myelin water fraction did show sensitivity to the ODCs. However, their analysis had several limitations, namely the use of a relatively coarse cortical atlas, cell type-specific gene expression lists based on mouse tissue rather than human tissue, and only male participants.

Genetic correlates of ${R_{2}}^{\ast }$ at 3T were investigated in Wen et al. (2018). The authors found that after removing some vascular-related MRI signal contributions, the spatial distribution of ${R_{2}}^{\ast }$ values were associated with the distribution of genes with ontologies related to neurons, glia (including astrocytes, microglia, and oligodendrocyte precursor cells [OPCs]), and endothelial cells. At 7T, ${R_{2}}^{\ast }$ has also been found to be associated with cytoarchitecture, specifically with neuronal cell counts from post-mortem atlases (McColgan et al. 2021).

In order to further investigate the biophysical inferences that are possible from the quantitative parameters ${R_{1}}$, MTsat, and ${R_{2}}^{\ast }$, in the following we examine associations of the parameters using a finer cortical atlas (Glasser et al. 2016) than Patel et al. (2020), and cell type-specific gene expression lists from human tissue (Habib et al. 2017, Hodge et al. 2019). The analysis is replicated using two cell type-specific gene expression lists to reduce the possibility that the results are dependent on a specific dataset. Because qMRI parameter contrast changes with the static magnetic field strength of the MRI scanner (Peters et al. 2007, Rooney et al. 2007, Wang et al. 2020), and this could potentially give rises to changes in sensitivity and specificity (Mancini et al. 2020), we investigate the associations at two different field strengths, 3T and 7T. To mitigate partial volume effects when examining the 1.6–4.5 mm thin cortex (Edwards et al. 2018), we exclusively use high, isotropic resolution data ($800 \mu $m at 3T and $500 \mu $m at 7T).

## Materials and methods

### MRI acquisition and preprocessing

3T acquisition: We used MPM data (Carey et al. 2018, Weiskopf et al. 2013) from 17 healthy volunteers (5 female, 12 male, mean age $\pm $ standard deviation: $29.2\pm 6.8$ years) from the MEG UK database (https://meguk.ac.uk/database), acquired on a 3T Prisma equipped with a 32-channel receive radiofrequency (RF) head coil (Siemens Healthineers, Erlangen, Germany) and a body RF transmit coil at the Wellcome Centre for Human Neuroimaging, UCL, London, following the same high resolution protocol as in Bonaiuto et al. (2018). The MPM protocol consisted of three RF- and gradient-spoiled, multi-echo 3D FLASH scans with PD-, T1-, and MT-weighting (PDw, T1w, and MTw) at $800 \mu $m isotropic resolution, plus a map of the RF transmit field $B_1$ acquired using a 3D-EPI spin echo/stimulated echo method (SE/STE) corrected for geometric distortions due to spatial inhomogeneities in the static magnetic field $B_0$ (Lutti et al. 2010). PDw: repetition time (TR) 25 ms; 8 equispaced echoes with echo time (TE) $[2.34,\ldots ,18.44]$ ms; flip angle (FA) 6$^{\circ }$. T1w: TR 25 ms; 8 equispaced echoes with TE $[2.34,\ldots ,18.44]$ ms; FA 21$^{\circ }$. MTw: TR 25 ms; 6 equispaced echoes with TE $[2.34,\ldots ,13.84]$ ms; FA 6$^{\circ }$; Gaussian RF magnetization transfer (MT) saturation pulse 2 kHz off resonance, 4 ms duration, nominal flip angle 220$^{\circ }$ prior to each FLASH excitation. Additional parameters: matrix size ($\textrm{read}\times \textrm{phase}\times \textrm{partition}$) $320\times 280\times 224$, GRAPPA (Griswold et al. 2002) $2\times 2$, non-selective sinc excitation, readout bandwidth 488 Hz/pixel.

7T acquisition: MPM data from 10 healthy volunteers (6 female, 4 male, $28\pm 3.6$ years) were acquired on a 7T whole-body MRI system (Magnetom 7T, Siemens Healthineers, Erlangen, Germany) equipped with a 1-channel transmit/32-channel receive RF head coil (Nova Medical, Wilmington, MA, USA) at the Max Planck Institute for Human Cognitive and Brain Sciences, Leipzig; these data were previously used in McColgan et al. (2021). The MPM protocol consisted of two RF- and gradient-spoiled, multi-echo 3D FLASH scans (PDw, T1w) adapted for whole-brain coverage at $500 \mu $m isotropic resolution (Trampel et al. 2019), plus a map of $B_1$ using a 3D-EPI SE/STE method adapted for 7T corrected for geometric distortions due to inhomogeneities in $B_0$ (Lutti et al. 2012). PDw: TR 25 ms, 6 equispaced echoes with TE $[2.8,\ldots ,16]$ ms, FA 5$^{\circ }$. T1w: TR 25 ms, 6 equispaced echoes with TE $[2.8,\ldots ,16]$ ms, FA 24$^{\circ }$. Additional parameters: matrix size ($\textrm{read}\times \textrm{phase}\times \text{fast/inner phase encode direction}$) $496\times 434\times 352$, GRAPPA (Griswold et al. 2002) $2\times 2$, non-selective sinc excitation, readout bandwidth 420 Hz/pixel. To mitigate the large $B_1$ inhomogeneity at 7T, two dielectric pads (Webb, 2011) were placed around the head of each subject (one each side) at approximately the level of the temporal lobe. The transmit voltage was calibrated to be optimal over the occipital lobe using an initial low-resolution transmit field map. For the purposes of prospective motion correction (Zaitsev et al. 2015), each subject was scanned while wearing a tooth clip assembly (molded to their front teeth) with an attached passive Moiré pattern marker (Vaculčiaková et al. 2022). An optical tracking system (Kineticor, Honolulu, HI, USA) tracked the motion of this marker (and thus motion of the head), allowing prospective rigid-body correction of the field of view.

The studies were approved by the local ethics committees and all subjects gave written informed consent before being scanned.

MRI data at each field strength were converted to qMRI maps of ${R_{1}}$, ${R_{2}}^{\ast }$, proton density (PD), and (at 3T only) MTsat using the hMRI toolbox (Tabelow et al. 2019, http://hmri.info). MTsat maps were not computed at 7T because specific absorption rate (SAR) limits at this field strength (Collins et al. 2004) hindered the acquisition of high-quality MTw images.

Cortical surfaces were reconstructed using the recon-all pipeline from FreeSurfer (Fischl et al. 2004, https://surfer.nmr.mgh.harvard.edu). Because the contrast in the 3T and 7T qMRI maps deviates significantly from the T1w MPRAGE image contrast expected by the recon-all pipeline (Carey et al. 2018), the following steps were taken to extract an image with MPRAGE-like contrast from the 3T and 7T qMRI parameters (McColgan et al. 2021). First, a small number of negative and very high values produced by estimation errors were set to 0 in the ${R_{1}}$ and PD maps, such that $T_1 (=1/R_{1})$ was bounded between $[0,8,000] \ {\textrm ms}$ and PD between $[0,200]\%$. Then, the PD and $T_1$ maps were used as input to the FreeSurfer mri_synthesize routine to create a synthetic FLASH volume with optimal white matter (WM)/grey matter (GM) contrast (TR 20 ms, FA 30$^{\circ }$, TE 2.5 ms). This synthetic image was used as the input to SPM segment (https://www.fil.ion.ucl.ac.uk/spm) to create a combined GM/WM/cerebrospinal fluid (CSF) brain mask (threshold: $\textrm{tissue probability}>0$), which was used for skull stripping.

For the 3T MPMs, the skull-stripped synthetic image was then used as input for the remaining steps of the recon-all pipeline to reconstruct cortical surfaces.

At 7T, using the skull-stripped synthetic T1w image as input to FreeSurfer frequently led to errors in the recon-all pipeline (McColgan et al. 2021). Figs. S3 and S4 in the Supplementary Material imply that this is likely because localised artefacts in the 7T ${R_{1}}$ map propagate to the synthetic T1w image. Thus, at 7T the PD map (corrected for spatial bias and normalised such that the average WM intensity is 69% as part of the standard hMRI toolbox pipeline (Tabelow et al. 2019)) was subtracted from 100% (i.e. the contrast was inverted) to yield a $(1-\textrm{PD})$ map (Mezer et al. 2013), which had MPRAGE-like contrast. This $(1-\textrm{PD})$ map was then denoised (Maggioni et al. 2013, http://www.cs.tut.fi/∼foi/GCF-BM3D) to mitigate the increased noise levels in the higher resolution 7T data compared to 3T, and the brain mask from the synthetic image was applied. The resulting denoised and masked $(1-\textrm{PD})$ map was then used in the recon-all pipeline to reconstruct cortical surfaces.

Examples of the input quantitative maps and synthesized images can be found in the Supplementary Material.

For both field strengths, cortical qMRI parameter values were mapped onto the surfaces from the recon-all pipeline using values sampled at 50% of the estimated vertex-wise cortical depth (i.e. we sampled at approximately the central cortical surface) and 2D-smoothed on the surface with a 6 mm full-width half-maximum (FWHM) kernel. This surface-based smoothing helps to mitigate any small errors in cortical layer segmentation and differences in the location of cortical areas between participants (Hagler et al. 2006). Finally, FreeSurfer was used to perform surface based registration of the HCP-MMP1.0 cortical atlas (Glasser et al. 2016) from fsaverage template space (Mills, 2016) to subject space (Neurolab, 2018).

### Cell type-specific gene expression analysis

The cell type-specific gene expression analysis proceeded in two steps, described in detail below. In the first step, we constructed target gene lists from the genes with the strongest spatial associations between the AHBA gene expression data and each of the qMRI parameters using partial least squares (PLS) regression. The second step examined whether these target genes were expressed more than expected by chance within particular cell types using the Expression Weighted Cell type Enrichment (EWCE) toolbox (Skene & Grant, 2016). A flow chart showing this procedure can be found in the Supplementary Material (Fig. S5).

The AHBA of gene expression (Hawrylycz et al. 2012) was mapped into the 180 parcellation units of the left hemisphere of the HCP-MMP1.0 atlas (Glasser et al. 2016) by following steps 1–6 in Arnatkevi˘ciūtė et al. (2019) using code available at https://github.com/BMHLab/AHBAprocessing to give a $(\textrm{gene}\times \text{region of interest (RoI)})$ matrix. The code was run using the options recommended by Arnatkevi˘ciūtė et al. (2019). Only left hemisphere data are presented as right hemisphere data are not available for all AHBA donors. Three areas in the HCP-MMP1.0 atlas – retroinsular cortex, middle temporal area, and area anterior 10p (Glasser et al. 2016) – did not robustly contain samples in the AHBA and were thus omitted from further analyses. This resulted in a $(\textrm{gene}\times \textrm{RoI})$ matrix of size $10,027\times 177$.

Each qMRI parameter at each field strength was averaged within each parcellation unit of the left hemisphere of the HCP-MMP1.0 atlas defined in fsaverage space (Mills, 2016), and also over subjects, resulting in an $(\textrm{RoI}\times \textrm{qMRI parameter})$ vector of size $177\times 1$ (for ${R_{1}}$ at 7T $175\times 1$; see below). Each vector was standardized by subtracting the mean and dividing by the standard deviation over the elements in the vector before further analysis. Dimensional reduction was performed separately for each qMRI parameter using PLS regression (Abdi, 2010, Krishnan et al. 2011, Rosipal et al. 2006) into the two PLS components which explained the most covariance between the spatial distribution of the genes and the spatial distribution of the qMRI parameter. The predictor variable in each case comprised the $(\textrm{gene}\times \textrm{RoI})$ matrix, and the response variable the $(\textrm{RoI}\times \textrm{qMRI parameter})$ vector. Each step of PLS was performed using the plsregress function in *Matlab* (Mathworks, Natick, US-MA). Weights representing the contribution of each gene to each PLS component were estimated using the bootstrapping procedure described in Vértes et al. (2016) with 10,000 bootstrapped samples. Target lists representing the top 5%, 10%, and 20% of genes most positively associated (upweighted) and most negatively associated (downweighted) with each qMRI parameter were then created from these weights. We examined upweighted and downweighted associations separately to avoid potentially masking cell-type associations.

We report the estimate of the percentage variance explained in each of the original (i.e. non-bootstrapped) matrices and vectors by the PLS components as output by plsregress. This gives an estimate of the spatial variance explained in the spatial qMRI parameter and gene expression distributions by each PLS component. We only investigated components explaining $>10\%$ of the variance in each $(\textrm{RoI}\times \textrm{qMRI parameter})$ vector further.

To check whether our results were dependent on the cell type-specific gene sets used, we performed the further analysis steps using human-derived cell type-specific gene sets from two independent sources. Both of these datasets used RNA sequencing (RNA-seq) methods, giving sufficient dynamic range for EWCE analysis (Skene & Grant, 2016).

The first is the SMART-seq dataset (Hawrylycz et al. 2015, Hodge et al. 2019), which was downloaded from the AIBS Brain Map website (https://portal.brain-map.org/atlases-and-data/rnaseq; Multiple Cortical Areas - SMART-seq (2019)). These gene sets comprise gene expression sampled in cells belonging to the major cell types: astrocytes, endothelial cells, GABAergic (inhibitory) neurons, glutamatergic (excitatory) neurons, microglia, pericyte cells, vascular and leptomeningeal cells (VLMCs), oligodendrocytes (ODCs), and oligodendrocyte precursor cells (OPCs) (Hodge et al. 2019).

The second is the DroNc-seq dataset from the Regev laboratory (Habib et al. 2017). This has slightly different cell categories as it was derived from different regions (in parentheses are the abbreviations used in the dataset): astrocytes (ASC), endothelial cells (END), GABAergic neurons, glutamatergic neurons from the prefrontal cortex (exPFC), granule neurons from the hippocampal dentate gyrus region (exDG), ODCs, OPCs, microglia (MG), pyramidal neurons from the hippocampal CA region (exCA), and neuronal stem cells (NSC).

It was shown in Hodge et al. (2019, Extended Data Fig. 5) that correspondence can be made between the labels in the two datasets, namely between the respective labels for astrocytes, microglia, endothelial cells, ODCs, OPCs, and GABAergic neurons, and between the SMART-seq glutamatergic neuron and the DroNc-seq exPFC neuron labels. As the exCA, exDG, and NSC categories from the DroNc-seq dataset and the pericyte and VLMC categories from the SMART-seq dataset do not have analogs in the other respective dataset, we do not explore the results involving these cell types in the main text. Those results can be found in the Supplementary Material (Figs. S6–S17).

The EWCE toolbox (https://github.com/NathanSkene/EWCE; version 1.2.0) was used to determine whether genes within the target lists from the PLS components of each qMRI parameter have higher expression within a particular cell type than expected by chance (Skene and Grant, 2016, Zarkali et al. 2020a,b). For a given cell type-specific dataset (here, either the SMART-seq or the DroNc-seq dataset), for each cell type, $c$, EWCE first computes the average expression of each gene in the cell type. A sum is then made over the average expression values within the gene list associated within a target list, $X$, to obtain a single EWCE value for each cell type, $\gamma (X,c)$. To test the statistical significance of this value, it is compared with values obtained for bootstrap target lists, $X^{\prime}$, using the genes indexed in the cell type-specific dataset. Each comparison was run with 100,000 bootstrap lists (controlling for transcript length and GC (guanine–cytosine) content (Skene & Grant, 2016)), and statistical significance for each comparison was set at a Benjamini–Hochberg false-discovery-rate (FDR) corrected $P < 0.05$. To check robustness, the comparisons were repeated for target lists comprising the top 5%, 10%, and 20% of genes associated with each parameter. Results are visualized as the number of standard deviations by which $\gamma (X,c)$ deviates from the mean over the bootstrapped samples, $\overline{\gamma (X^{\prime},c)}$ (Skene & Grant, 2016).

For brevity, when genes within a target list from a quantitative parameter have higher expression within a particular cell type than expected by chance (i.e. this higher expression is significant), we say that that cell type is “associated” with that quantitative parameter. We refer to associations as replicating at the “N% replication level” when there is significant overlap with genes enriched in a cell type at the top 5%-of-genes level in one dataset that replicates at an equal or lower N%-of-genes level in the other dataset, where N% is the lowest %-of-genes level at which the overlap replicates. We take an association with a cell type to be “robust” if it replicates at the 5% replication level. Note that differences in the effect size are to be expected between the two cell type-specific datasets as they are taken from different brain areas and used different sequencing methods.

Because the signs of the PLS weights are difficult to interpret for MRI metrics (Romero-Garcia et al. 2019), we treat associations with upweighted genes and with downweighted genes identically and do not try to interpret them in terms of positive or negative correlations.

${R_{1}}$ at 7T is affected by $B_1$ and $B_0$ inhomogeneities in the inferior temporal and frontal lobes (McColgan et al. 2021), as can be seen in Figs. S3 and S4 in the Supplementary Material. These inhomogeneities become more important at 7T because their causes ($B_1$ field-focusing in brain-sized objects (Hoult, 2000) and (dynamic) susceptibility-induced contributions to the local $B_0$ field (Stockmann & Wald, 2018, Van de Moortele et al. 2002)) both increase from 3T to 7T. We thus excluded data from two potentially strongly affected regions in the 7T ${R_{1}}$ analysis (the orbitofrontal complex and area TE2 anterior (Glasser et al. 2016), the two areas which had ${R_{1}}$ values which were more than three standard deviations away from the mean) to mitigate the potential influence of these artefacts on the results; in this case, we thus used a $(\textrm{gene}\times \textrm{RoI})$ matrix of size $10,027\times 175$ and an $(\textrm{RoI}\times \textrm{7T} \ {{\textrm R}_{1}})$ vector of size $175\times 1$. The location of the omitted areas is shown in Fig. S18 in the Supplementary Material. Results where these two areas were included can be found in Table S1 and Figs. S19–21 in the Supplementary Material.

## Results

**Fig. 1:**
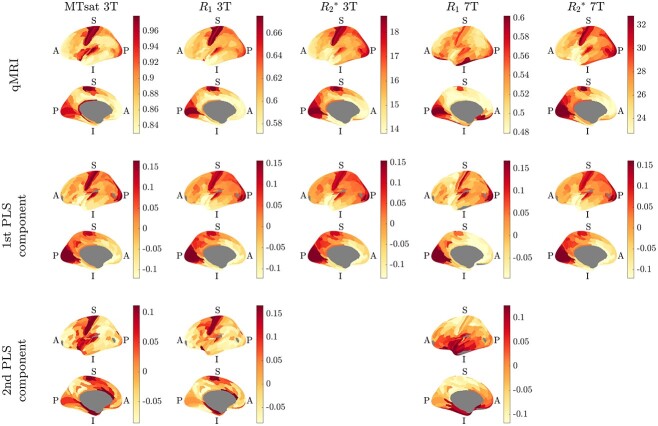
The left hemisphere spatial distribution of the qMRI parameters at each magnetic field strength (top row) and of the respective first and second PLS components (bottom two rows) projected on the inflated FreeSurfer fsaverage brain. The qMRI parameter plots show the mean over vertices and subjects in each area of the HCP-MMP1.0 atlas (units: MTsat$/ {\text{p.u.}}$; ${R_{1}}/{\textrm s}^{-1}$; ${R_{2}}^{\ast }/{\textrm s}^{-1}$). The PLS component plots show the score-vectors (Rosipal et al. 2006) of the $(\textrm{gene}\times \textrm{RoI})$ matrix for each qMRI parameter, giving a visual representation of the latent PLS variables (in arbitrary units). PLS components are only plotted when they explain $>10\%$ of the spatial variance of a qMRI parameter (Table 1). In each case, top: lateral view, bottom: medial view. A: anterior, P: posterior, I: inferior, S: superior. The regions marked in grey represent areas with no data, i.e. non-cortical tissue (mostly corpus callosum), regions without robust cortical samples in the AHBA, and the potentially artefact affected areas in the 7T ${R_{1}}$ case. Lower and upper limits of the colour maps in each plot are the 5th and 95th percentiles of the data, respectively. Colours from http://colorbrewer.org by Cynthia A. Brewer, Geography, Pennsylvania State University via https://github.com/DrosteEffect/BrewerMap.

The spatial distributions of the quantitative parameters averaged over subjects at each magnetic field strength are shown in Fig. 1. Primary cortical areas are clearly delineated, and subtle differences can be seen between the parameters, especially towards the posterior of the brain, around the superior temporal lobe, and (for ${R_{1}}$ at 7T) around the central sulcus. Some of the differences between 3T and 7T ${R_{1}}$ around the inferior frontal and temporal lobes could be due to the influence of the artefacts in the 7T ${R_{1}}$ maps (see the Data quality assessment in the Supplementary Material), justifying our decision to remove the most likely affected areas when analysing this parameter.

The spatial distribution of the PLS components in Fig. 1 shows a lot of similarity to the qMRI parameters, implying that we are reasonably capturing the spatial variance. The first component is very similar between all qMRI parameters, but with specific differences seen around the central sulcus in ${R_{1}}$ at 7T, in line with the different spatial distribution of the parameter in this region. The second component (plotted when the variance explained in the qMRI parameter was $>10\%$) captures more of the differences between the spatial distributions of the qMRI parameters.

Table 1 shows that the first PLS component explained more than 50% of the spatial variance of ${R_{1}}$ at 3T and of ${R_{2}}^{\ast }$ at both 3T and 7T, but less than 50% of the spatial variance of ${R_{1}}$ at 7T or of MTsat at 3T. For ${R_{1}}$ at 3T and 7T and MTsat at 3T the second PLS component explained more than 10% of the spatial variance and was therefore included in the further analysis.

**Table 1:** Variance explained by the PLS components for each qMRI parameter.

	PLS	Spatial variance explained in	
	component	gene distribution	qMRI parameter
$R_{2}^{\ast }$ 3T	1	22%	75%
	2	7%	6%
$R_{2}^{\ast }$ 7T	1	22%	71%
	2	9%	6%
MTsat 3T	1	21%	33%
	2	9%	21%
$R_{1}$ 3T	1	22%	60%
	2	7%	11%
$R_{1}$ 7T	1	17%	18%
	2	9%	20%

**Fig. 2:**
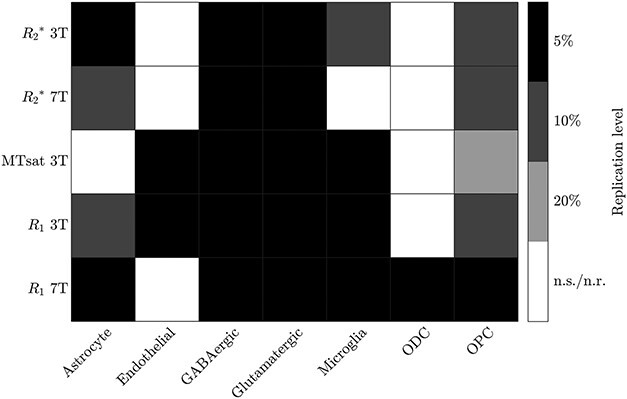
Summary of the significant, replicated associations found between cell type-specific gene expression in the genes associated with each qMRI parameter. Replications at the level of the top 5% of genes associated with each qMRI parameter (robust associations) are shown in black, with replications at lower levels in shades of grey. Non-significant (n.s.) and non-replicating (n.r.) associations are in white.

**Fig. 3:**
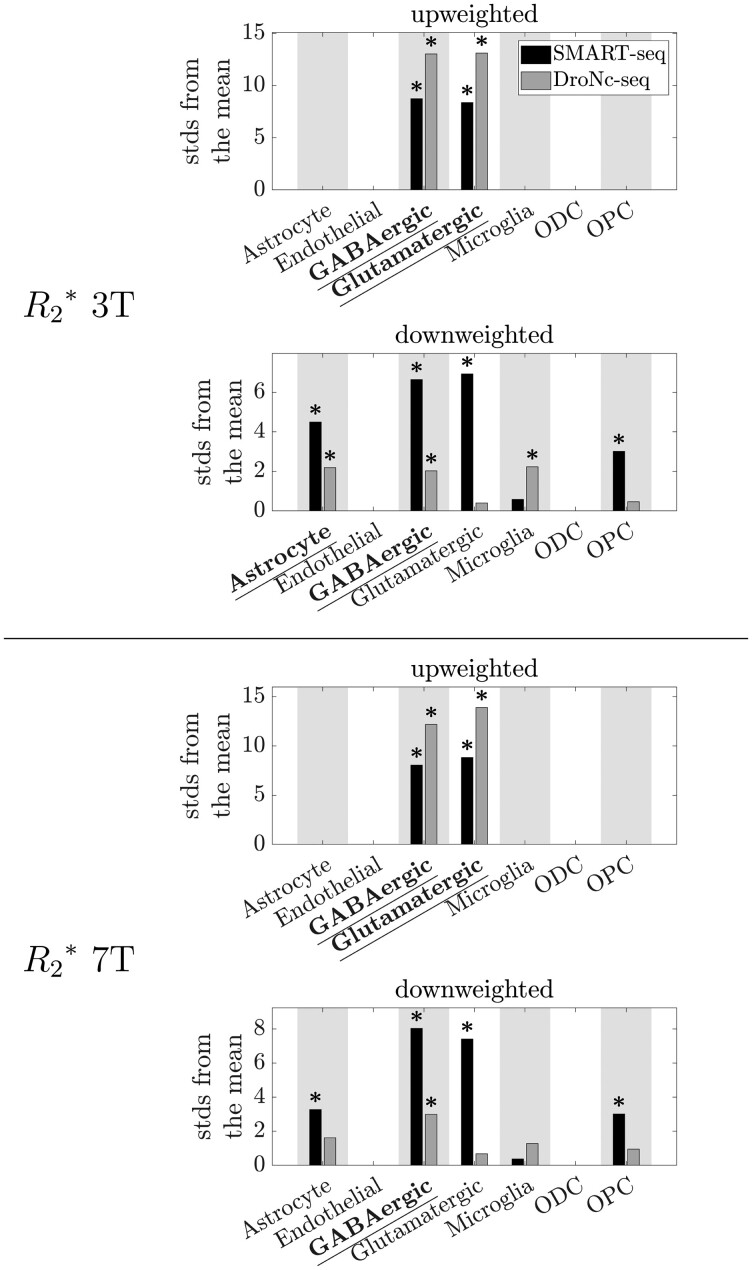
EWCE results showing the cell type associations of the top 5% of genes associated with ${R_{2}}^{\ast }$ at 3T and 7T (first PLS component only). Plotted are the number of standard deviations (stds) by which the EWCE value deviated from the mean value over bootstrapped target lists. Results from the two cell type-specific datasets are plotted in different colors: SMART-seq in black, DroNc-seq in grey. Top: 3T. Bottom: 7T. Bars are only plotted when FDR-corrected $P<0.5$. *: FDR-corrected $P<0.05$. Significant cell-type associations which replicated between both cell type-specific datasets (robust results) are underlined and in bold.

**Fig. 4:**
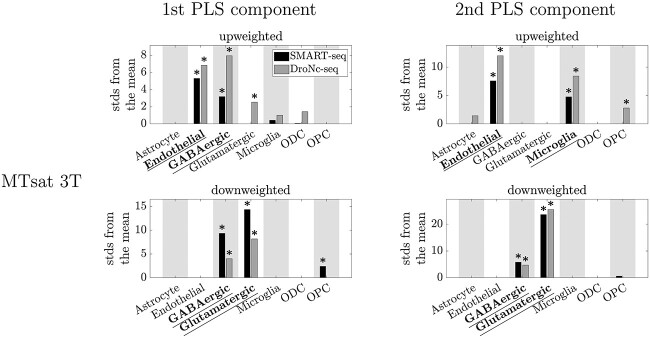
EWCE results showing the cell-type associations of the top 5% of genes associated with MTsat at 3T. Plotted are the number of standard deviations (stds) by which the EWCE value deviated from the mean value over bootstrapped target lists. Results from the two cell type-specific datasets are plotted in different shades: SMART-seq in black, DroNc-seq in grey. Left: First component of the PLS. Right: Second component of the PLS. Bars are only plotted when FDR-corrected $P<0.5$. *: FDR-corrected P$<0.05$. Significant cell-type associations that replicated between both cell type-specific datasets (robust results) are underlined and in bold.

**Fig. 5:**
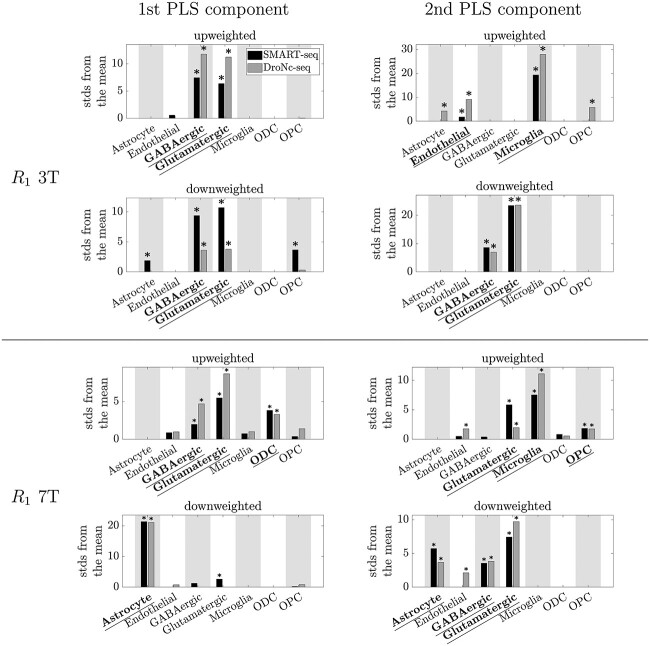
EWCE results showing the cell-type associations of the top 5% of genes associated with ${R_{1}}$ at 3T and 7T. Plotted are the number of standard deviations (stds) by which the EWCE value deviated from the mean value over bootstrapped target lists. Results from the two cell type-specific datasets are plotted in different shades: SMART-seq in black, DroNc-seq in grey. Top: 3T. Bottom: 7T. Left: First component of the PLS. Right: Second component of the PLS. Bars are only plotted when FDR-corrected $P<0.5$. *: FDR-corrected $p<0.05$. Significant cell-type associations which replicated between both cell type-specific datasets (robust results) are underlined and in bold.

The EWCE analysis results are summarised in Fig. 2 and detailed for each qMRI parameter separately in Figs. 3–5. The robust cell type-associations are shown in black in Fig. 2. In the following we go through the results for each qMRI parameter in turn.

At 3T ${R_{2}}^{\ast }$ showed robust associations with astrocytes, GABAergic neurons, and glutamatergic neurons (Figs. 2 and 3). There was also a significant association at the top 5% level with microglia in the DroNc-seq dataset and with OPCs in the SMART-seq dataset, but these each only replicated at the top 10% level in the other dataset (Fig. 2, Fig. S7, and S10).

At 7T the ${R_{2}}^{\ast }$ results were similar to those at 3T (Figs. 2 and 3). Robust associations were seen with GABAergic and glutamatergic neurons. Significant associations with astrocytes and OPCs were seen at the top 5% level in the SMART-seq dataset, but these only replicated at the top 10% level in the DroNc-seq dataset (Figs. 2, 3, and S10).

MTsat showed robust associations with endothelial cells, GABAergic neurons, glutamatergic neurons, and microglia (Figs. 2 and 4). A significant association was seen with OPCs at the top 5% level in the SMART-seq dataset for the first PLS component and in the DroNc-seq dataset for the second PLS component, but these results only replicated at the top 20% level in the respective other dataset (Figs. S11, and S14).

At 3T ${R_{1}}$ showed robust associations with endothelial cells, GABAergic neurons, glutamatergic neurons, and microglia (Figs. 2 and 5), showing some similarity to the MTsat results (Fig. 4). Significant associations were also seen with astrocytes and OPCs at the top 5% level in the SMART-seq dataset for the first PLS component, but these only replicated at the top 10% (Fig. S10) and top 20% (Fig. S11) levels in the DroNc-seq dataset, respectively. Similarly, associations with astrocytes and OPCs were also seen at the top 5% level in the DroNc-seq dataset for the second PLS component, but these only replicated at the top 20% (Fig. S14) and top 10% (Fig. S13) levels, respectively, in the SMART-seq dataset.

The 7T ${R_{1}}$ associations differed from the 3T results. Robust associations were seen with astrocytes, GABAergic neurons, glutamatergic neurons, microglia, ODCs, and OPCs (Figs. 2 and 5).

The results when including regions in the 7T ${R_{1}}$ analysis that were potentially strongly affected by $B_1$ and $B_0$ artefacts (the orbitofrontal complex and area TE2 anterior (Glasser et al. 2016)) can be found in the Supplementary Material. Including these regions resulted in a slight increase in the variance explained in ${R_{1}}$ by the first PLS component, but a larger decrease in the variance explained by the second PLS component (Table S1), such that overall the variance explained in ${R_{1}}$ by the two components decreased. There were some changes in the cell-type associations (Fig. S19): a robust association with genes enriched in GABAergic neurons was only observed in the second PLS component, rather than in both PLS components, and the robust association with genes enriched in OPCs was not present (cf. Figs. 2 and 5). A further association with endothelial cells was also seen at the top 5% level in the DroNc-seq dataset, which only replicated at the top 20% level in the SMART-seq dataset (see the full set of results in Figs. S20 and S21).

## Discussion

The EWCE results showed robust associations of excitatory and inhibitory neurons with all qMRI parameters under consideration at both 3T and 7T, implying that neurons are (i.e. cytoarchitecture is) the main predictor of these cortical qMRI contrasts. This observation is in line with previous observations of general cortical gradients between sensorimotor and higher areas in many different modalities (Huntenburg et al. 2018), which are also visible in Fig. 1. In addition, ${R_{2}}^{\ast }$ at 3T showed robust associations with astrocytes; MTsat at 3T showed robust associations with endothelial cells and microglia; ${R_{1}}$ at 3T showed robust associations with microglia and endothelial cells; and ${R_{1}}$ at 7T showed robust associations with microglia, ODCs, OPCs, and astrocytes.

Fig. 2 gives a visual impression of the associations. It shows that while the qMRI parameters are highly correlated – as would be expected due to their dependence on the same underlying biological substrate – they are not identical in their associations. The differential associations of the spatial distribution of the qMRI parameters with different cell types implies that by combining them, we could become sensitive to specific cell types. As an example, combination of ${R_{2}}^{\ast }$ at 3T and at 7T could potentially allow inference of the spatial distribution of astrocytes, as despite otherwise similar associations, ${R_{2}}^{\ast }$ at 3T is robustly associated with astrocytes, but ${R_{2}}^{\ast }$ at 7T is not. This presents an interesting direction for future research. Fig. 2 is intended to allow other such parameter combinations to be easily read off.

### ${{\boldsymbol R}_{\boldsymbol 2}}^{\ast }$ associations

The regional distribution of ${R_{2}}^{\ast }$ in the cortex was robustly associated with excitatory (glutamatergic) and inhibitory (GABAergic) neurons, and this association was very consistent between field strengths. This association is in line with the observations by McColgan et al. (2021) at 7T (who linked cytoarchitecture from post-mortem histology to the same 7T data as used here) and by Wen et al. (2018) at 3T, providing further evidence that cortical ${R_{2}}^{\ast }$ is sensitive to neuron density (Zhao et al. 2016). The relationship to cytoarchitecture suggests an indirect link to myelin, as local neurons are both the source of local myelinated axons (Braitenberg, 1962, Dinse et al. 2015, Hellwig, 1993, Micheva et al. 2018), and their dendrites are the target of remote axons entering the cortex. However the existence of general cortical gradients (Huntenburg et al. 2018) make the direction of the effect difficult to determine.

We did not see robust associations of ${R_{2}}^{\ast }$ with endothelial cells or some of the glia types (microglia and OPCs) observed by Wen et al. (2018), though we did see an association with astrocytes at 3T, which could reflect the sensitivity of ${R_{2}}^{\ast }$ to iron (Edwards et al. 2018, Möller et al. 2019). We note, however, that associations with microglia (3T) and OPCs (3T and 7T) were each significant in one of the two cell type-specific datasets (Fig. 2).

### MTsat associations

MTsat is commonly interpreted as representing macromolecular content, as it cleanly differentiates between GM, WM, and cerebrospinal fluid (Callaghan et al. 2015, Helms et al. 2008) and correlates with post mortem tissue metrics of myelin (Georgiadis et al. 2021), one of the largest sources of macromolecules in the brain. Interestingly, MTsat did not show robust associations with ODCs or OPCs (which would represent a direct relation to myelin), but was robustly associated with genes enriched in excitatory and inhibitory neurons, i.e. cytoarchitecture, like ${R_{2}}^{\ast }$, suggesting a potential indirect link to myelin. It should also be noted that there is significant cortical macromolecular content that is not associated with myelin (Mezer et al. 2013), which would not be captured by ODC and OPC associations.

The robust association of MTsat with genes enriched in endothelial cells suggests an association with cortical vasculature, as these cells line the walls of blood vessels (Duvernoy et al. 1981). A relationship between MTsat and vasculature is surprising when one considers the interpretation of MTsat as a myelin marker. One possible explanation is that the macromolecules in the endothelial cells could give rise to an observable MT effect. Another explanation could be a mechanism of magnetization transfer studied in the context of functional MRI (Kim et al. 2008, Pike et al. 1992, Schulz et al. 2020). In short, off-resonance MT-saturation pulses can efficiently saturate the water spins in cortical tissue, but not those in blood. Perfusion of this non-saturated blood into the saturated tissue via capillaries will give a local increase in signal, with the amount of perfused blood and thus the signal increasing proportionally to the amount of local vascularisation. As the amount of local vascularisation is spatially varying (e.g. primary cortical areas have a highly vascularised layer IV (Schmid et al. 2019)), this could give rise to spatial variance in MTsat, explaining the observed relationship between MTsat and endothelial cells. Relatedly, the robust association of MTsat with microglia could be due to “off-resonance saturation” (Bossoni et al. 2022, Delangre et al. 2015, Zurkiya & Hu, 2006) in the neighborhood of iron-rich microglia. Off-resonance saturation has been shown to be additive to the MT effect (Delangre et al. 2015, Zurkiya & Hu, 2006).

### ${{\boldsymbol R}_{\boldsymbol 1}}$ associations

Like for ${R_{2}}^{\ast }$, the regional distribution of ${R_{1}}$ at both 3T and 7T was robustly associated with genes enriched in excitatory (glutamatergic) and inhibitory (GABAergic) neurons. The similarity of the ${R_{1}}$ associations to those of ${R_{2}}^{\ast }$ was particularly strong at 3T (compare Figs. 3 and 5).

In addition to the neuronal associations, ${R_{1}}$ at 3T was robustly associated with gene expression associated with endothelial cells and microglia, showing similarity to the MTsat results. A similarity between ${R_{1}}$ and MTsat results is expected because of MT contributions to ${R_{1}}$ modulated by the excitation pulse (Olsson et al. 2020, Teixeira et al. 2019). However, the associations could alternatively be due to iron-induced contributions (Rooney et al. 2007, Stüber et al. 2014) to the ${R_{1}}$ (Möller et al. 2019).

${R_{1}}$ at 7T showed, in addition to the neuronal associations, robust associations with genes enriched in astrocytes and microglia, which could, like similar associations at 3T, reflect iron-induced contributions to the relaxation (Möller et al. 2019); the lack of the astrocyte association at 3T and the endothelial cell association at 7T could suggest a magnetic field strength dependence of the relaxation contributions from these cell types. It should be noted, however, that the magnetic field strength dependence of iron contributions to ${R_{1}}$ is expected to be small (Rooney et al. 2007, Wang et al. 2020).

The regional distribution of ${R_{1}}$ at 7T was also robustly associated with genes enriched in ODCs and OPCs. This is consistent with the use of 7T ${R_{1}}$ as a cortical myelin marker (as reviewed in Edwards et al. (2018)), though it should be noted that ODCs are also iron rich (Möller et al. 2019), and myelin and iron concentration are correlated (Fukunaga et al. 2010, Kirilina et al. 2020).

Our observation of a robust association of ${R_{1}}$ at 7T with ODCs and OPCs suggests that ${R_{1}}$ at 7T could be more sensitive to myelin than at 3T. This appears to contradict the results of Rooney et al. (2007) and Wang et al. (2020), who found that the contribution of myelin to ${R_{1}}$ decreases going from 3T to 7T. However the analysis of Wang et al. (2020) did show that going from 3T to 7T increases the MT with the macromolecular pool; this MT increase could explain the increased apparent myelin sensitivity, as our ${R_{1}}$ estimates are affected by MT (Olsson et al. 2020, Teixeira et al. 2019). It should be noted that this assessment is somewhat contradicted by the MTsat results (Fig. 4) being more similar to the 3T than the 7T ${R_{1}}$ results (Fig. 5).

The observation of a robust association of ${R_{1}}$ with endothelial cells at 3T but not at 7T could be due to relative changes in the ${R_{1}}$ of grey matter and venous blood with magnetic field strength, which, based on literature values of the respective ${R_{1}}$ values (Deistung et al. 2008), would lead to a relative decrease in the contribution of blood going from 3T to 7T. However, we cannot rule out that it could also be due to differences in the contribution of flow artefacts. These are likely to be more prevalent in the 7T data because while at 3T the scanner’s RF body coil was used for excitation, giving spatially non-selective spin excitation over a large region, at 7T a head-only RF transmit coil was used, meaning spin excitation was more localised. The localised transmission means that spins in the blood flowing into the brain are not excited: the effects of in-flow from these non-excited spins could blur the image contrast by giving rise to a spatially differentiated increase in physiological noise correlated with spatial variations in cortical vascularisation.

It is of interest to note that the spatial distribution of the score vectors of the $(\textrm{gene}\times{}\textrm{RoI})$ matrix of the first and second PLS components of ${R_{1}}$ and MTsat at 3T show similar spatial patterns (Fig. 1), following the general gradient observed in neuroimaging (Goulas et al. 2021, Huntenburg et al. 2018). This stands in contrast to the score vector of the second PLS component of the ${R_{1}}$ at 7T, which is visibly different with an apparent superior–inferior gradient. The difference in the spatial distributions of the second PLS component could give clues to the source of the differences, but could also be a result of this component reflecting stronger $B_1$ and $B_0$ artefacts at 7T compared to 3T (Hoult, 2000, Stockmann & Wald, 2018, Van de Moortele et al. 2002).

To mitigate the possibility that artefacts at 7T affect our results, we performed the 7T ${R_{1}}$ analysis after removing regions expected to be potentially affected. It can be seen in Table S1 in the Supplementary Material that if these areas are included in the 7T ${R_{1}}$ analysis, then the variance explained by the first two PLS components decreases, suggesting that including these areas introduces a source of variance that cannot be explained using the gene expression distribution, i.e. that these areas were affected by artefacts. However, the only difference in robust associations if these areas are included is that the 7T ${R_{1}}$ would no longer be robustly associated with genes enriched in OPCs. As ${R_{1}}$ at 7T is still associated with ODCs even with these areas included, including them would not have changed our assessment that ${R_{1}}$ at 7T is more closely associated with cell types strongly related to myelination than the other quantitative parameters that we investigated.

The 3T results for MTsat and ${R_{1}}$ deviated from the results for MTR and ${R_{1}}$ in Patel et al. (2020). As the 3T dataset used here gave results comparable to Patel et al. (2020) when using a similar pipeline to the one they used (Edwards et al. 2019), we can attribute the discrepancy to the different analysis pipeline used here. The two major differences (although other differences such as gene normalisation could also play a role (Markello et al. 2021)) were that we used (1) a finer cortical atlas (180 cortical areas vs. 34) and (2) human-derived gene lists (Habib et al. 2017, Hodge et al. 2019) rather than mouse-derived lists (Zeisel et al. 2015).

(1) The sampling density of gene expression over the cortical surfaces in the AHBA is relatively sparse, and so averaging expression levels over regions of interest (RoIs) helps to increase the robustness of the results (Arnatkevi˘ciūtė et al. 2019). If the RoIs are too large, however, then spatial specificity is lost, as functionally and anatomically distinct cortical areas get merged together. The HCP-MMP1.0 atlas is derived based on boundaries found from in vivo anatomical and functional MRI data (Glasser et al. 2016), allowing reasonable specificity, while the RoIs are sufficiently large that a reasonable mapping to the gene expression samples in the AHBA atlas is possible (Arnatkevi˘ciūtė et al. 2019).

(2) Mouse-derived cell type-specific gene expression has been found to be less able to discriminate cell types in human because of species-specific features, especially for non-neuronal cell types (Hodge et al. 2019). Human-derived lists should thus be preferred where possible.

### Limitations

The lack of robust associations of ${R_{2}}^{\ast }$ with endothelial cells, microglia and OPCs is potentially because our choice of echo times and, relatedly, our choice of algorithm to estimate ${R_{2}}^{\ast }$ differed significantly from that in Wen et al. (2018). Wen et al. (2018) used the extensive range of echo times in their MRI protocol (10 equispaced echoes from 4 to 40 ms) to separate the signal decay into fast relaxing (interpreted as vascular) and slow relaxing (interpreted as tissue) components (Ulrich & Yablonskiy, 2016). Our echo times were more limited (8 equispaced echoes from 2.34 to 14.1 ms at 3T and 6 equispaced echoes from 2.8 to 16 ms at 7T), and further our data were recorded at higher resolution, and thus had a lower signal-to-noise ratio (SNR). To mitigate both of these factors, we assumed a common monoexponential ${R_{2}}^{\ast }$ decay between PDw, T1w, and MTw images (Weiskopf et al. 2014) to allow robust estimation of ${R_{2}}^{\ast }$. Our assumption of single exponential decay will mix decay rates of the slow and fast relaxing components, with a relative weighting towards the faster component because of our lower maximal echo times. On top of this, the assumption of a common ${R_{2}}^{\ast }$ decay between PDw, T1w, and MTw images can break down in complex multi-compartment systems like brain tissue (Chan & Marques, 2020). Unfortunately, neither our 3T nor our 7T protocol allows us to apply the algorithm used by Wen et al. (2018) to explore this further.

We did not include any effects of orientation with respect to the scanner’s static magnetic field in our analysis. ${R_{2}}^{\ast }$ and MTR (a parameter related to MTsat) have been shown previously to exhibit a dependence on the orientation of the cortex with respect to the magnetic field (Cohen-Adad et al. 2012, Mangeat et al. 2015). ${R_{1}}$ could also be orientation dependent in line with observations of anisotropy in white matter (Knight et al. 2018, Schyboll et al. 2020). The orientation dependence of these parameters is fundamentally due to the regular structure of the microscopic myelin distribution in cortex, with most myelinated axons running either tangentially or radially with respect to the cortical surface (Nieuwenhuys et al. 2015, Vogt and Vogt, 1919). For ${R_{2}}^{\ast }$, the anisotropy of the myelin distribution propagates to the quantitative parameter through the anisotropic susceptibility distribution of myelin (Bender & Klose, 2010, Cohen-Adad et al. 2012). As this mechanism depends on susceptibility, we would expect the orientation dependence to scale with field strength. However, the similarity of our ${R_{2}}^{\ast }$ results at 3T and 7T would suggest that orientation dependence does not play a major role, likely because the averaging over cortical areas also averages over cortex oriented at a range of angles to the magnetic field.

The first PLS component explained the majority of the variance in the spatial distribution of ${R_{2}}^{\ast }$ at 3T and 7T and ${R_{1}}$ at 3T, while a second PLS component was additionally needed to explain the majority of the variance in MTsat. In the case of ${R_{1}}$ at 7T, however, the explained variance was still not over 50%, implying that there could be major sources of variance (e.g. additional cell types or imaging artefacts) that are important for the spatial contrast distribution in this case which are not included in our model. We mitigated one potential source of variance that would not be explicable in terms of cell types by removing potentially strongly artefact-affected areas from the 7T ${R_{1}}$ analysis. Including these areas would have decreased the variance explained even further (compare Tables 1 and S1).

We only examined associations with MRI parameters sampled on the central cortical surface. This choice was made to exclude as far as possible the contribution of partial volume effects with the white matter and CSF when comparing between the 3T and 7T data, and thus mitigate any confounding effects from the lower resolution of the 3T data. Our previous work using the 7T data presented here has shown that across the depth of the cortex ${R_{2}}^{\ast }$ (but not ${R_{1}}$) has strong associations with genes specific to cytoarchitectonic cortical layers II, III, IV, and V (McColgan et al. 2021).

Our test of the association of MPMs with cell types is indirect, relying on the cell type-specificity of genes. Future analyses could refine the analysis by including maps of neurotransmitter receptors (Dukart et al. 2021, Goulas et al. 2021), as these could give greater specificity when testing the associations with neurons.

Our in vivo data comes from young adults. In contrast, the post mortem gene expression atlases and cell type-specific gene expression datasets come from donors with a broader range of ages, most of which are older than our subjects (Habib et al. 2017, Hawrylycz et al. 2015, Hodge et al. 2019). During the mapping of gene expression from the AHBA donor datasets to the HCP-MMP1.0 atlas (Arnatkevi˘ciūtė et al. 2019), the genes were filtered based on differential stability to mitigate subject-specific effects (Hawrylycz et al. 2015). However, as the cortical cell distribution (e.g. of glia) is dynamic (Arnatkevi˘ciūtė et al. 2019, Edwards et al. 2018, Marsh et al. 2022), the regional gene expression atlas may not be entirely representative of our cohort. This could potentially affect the sensitivity of the method to individual cell types. It should be noted, though, that regional variation in cortical gene expression has previously been found to be relatively conserved between individuals (Hawrylycz et al. 2015).

Our results suggest that interareal-variations in MPMs largely reflect differences in gene expression associated with neurons, i.e. with cytoarchitecture. These results are however not necessarily applicable to longitudinal or inter-subject/-group comparisons, which can give rise to different associations. An example is provided by Patel et al. (2019): their gene expression analysis results showed that while the spatial distribution of MTR was not significantly associated with ODCs at either age 14 or 5 years later at age 19, the change in MTR between the two time points was significantly associated with ODCs. The results presented here imply that it would be interesting to examine such cases using the broad range of qMRI parameters and static magnetic field strengths examined here.

### Conclusions

The spatial distribution of all of the quantitative MRI parameters at both 3T and 7T robustly covaried with the distribution of genes enriched in neurons. This reflects the importance of cytoarchitecture in determining MRI contrast.

In addition to the general association with neurons, the spatial distribution of the parameters was found to robustly covary with the distribution of genes enriched in astrocytes (${R_{2}}^{\ast }$ at 3T, ${R_{1}}$ at 7T), endothelial cells (${R_{1}}$ and MTsat at 3T), microglia (${R_{1}}$ and MTsat at 3T, ${R_{1}}$ at 7T), and ODCs and OPCs (${R_{1}}$ at 7T). As the differences in spatial distributions of the parameters were associated with different cell types, these results imply it may be possible to extract information about individual cell types by combining the quantitative parameters.

The results complement the traditional interpretation of qMRI parameters in terms of iron and myelin, and advance the possible use of qMRI parameters as biomarkers for specific cell types, bringing us closer to the goal of in vivo histology using MRI.

## Supplementary Material

EdwardsQMRIgeneExpressionSuppInf_bhac453
