# Supplementary material for: Quantitative MRI maps of human neocortex explored using cell type-specific gene expression analysis
Source: Cereb Cortex. 2022 Dec 15;33(9):5704–16. doi: 10.1093/cercor/bhac453 (PMC10152104; doi:10.1093/cercor/bhac453)
Supplement: EdwardsQMRIgeneExpressionSuppInf_bhac453 [file edwardsqmrigeneexpressionsuppinf_bhac453.pdf]

Supplementary Material

Data quality assessment

Here we show examples of the quantitative MRI parameter maps, and the synthetic images derived from them that enter the cortical segmentation pipeline. The data were selected based on the standard-deviation-in-white matter ${R_2}^*$ (SD-${R_2}^*$) quality assurance metric (Castella et al., 2018) computed during the hMRI toolbox pipeline (Tabelow et al., 2019, Appendix D), where lower SD-${R_2}^*$ implies better data quality. We show as examples the subjects with the smallest and the largest root-mean-square SD-${R_2}^*$ over all weighted contrasts (PDw and T1w at 7T, and PDw, T1w and MTw at 3T) at each field strength. Please note that SD-${R_2}^*$ values are not directly comparable between field strengths because of differences in the number of echoes, contrasts, etc.

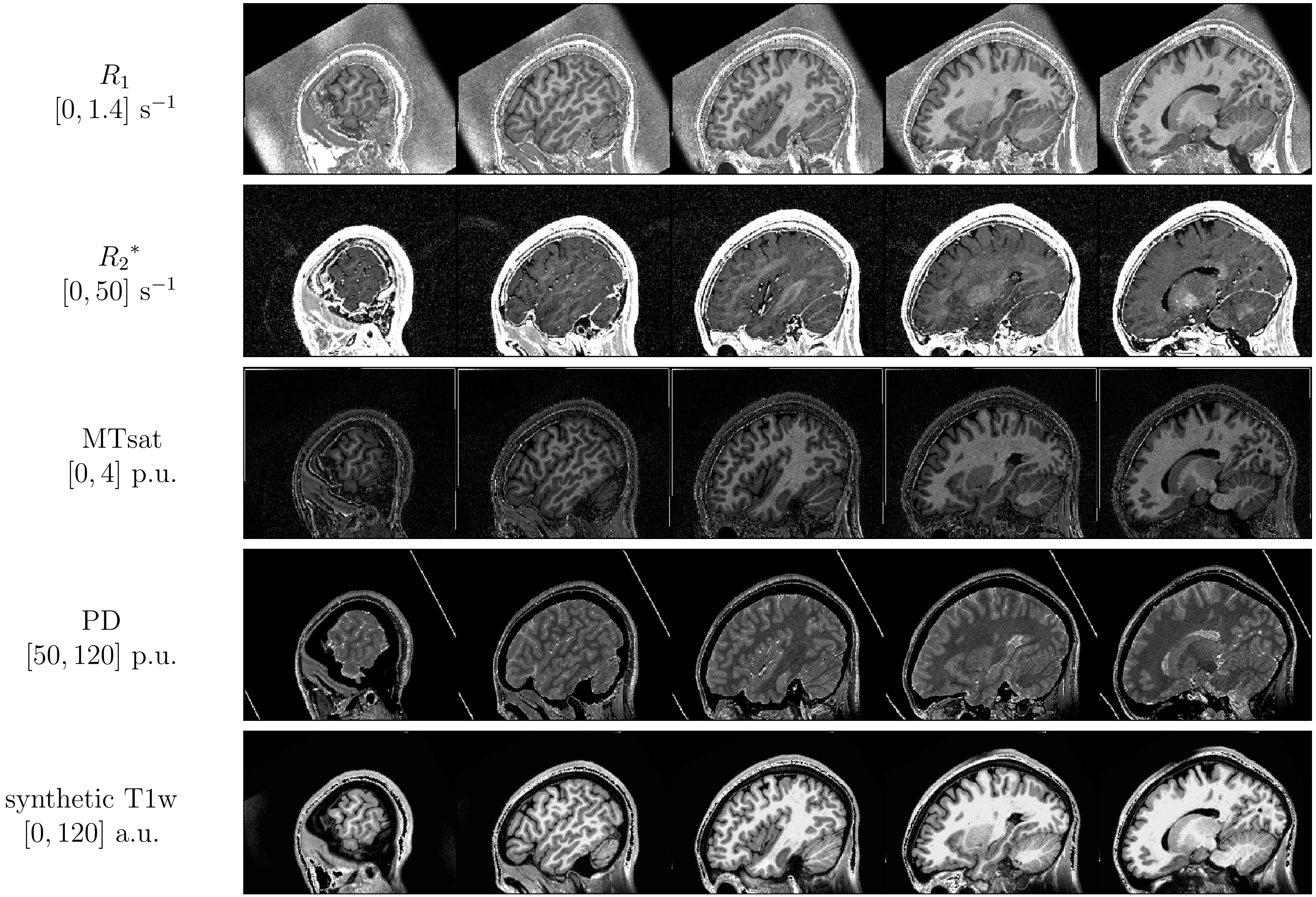

Figure S1: Sagittal slices of MPMs and synthetic images at 3T from the subject with the lowest SD-$R_2{}^*$, i.e. the best data quality. From left to right are slices of the left hemisphere going from lateral to medial. Only the left hemisphere is shown as only this hemisphere was investigated in this study.

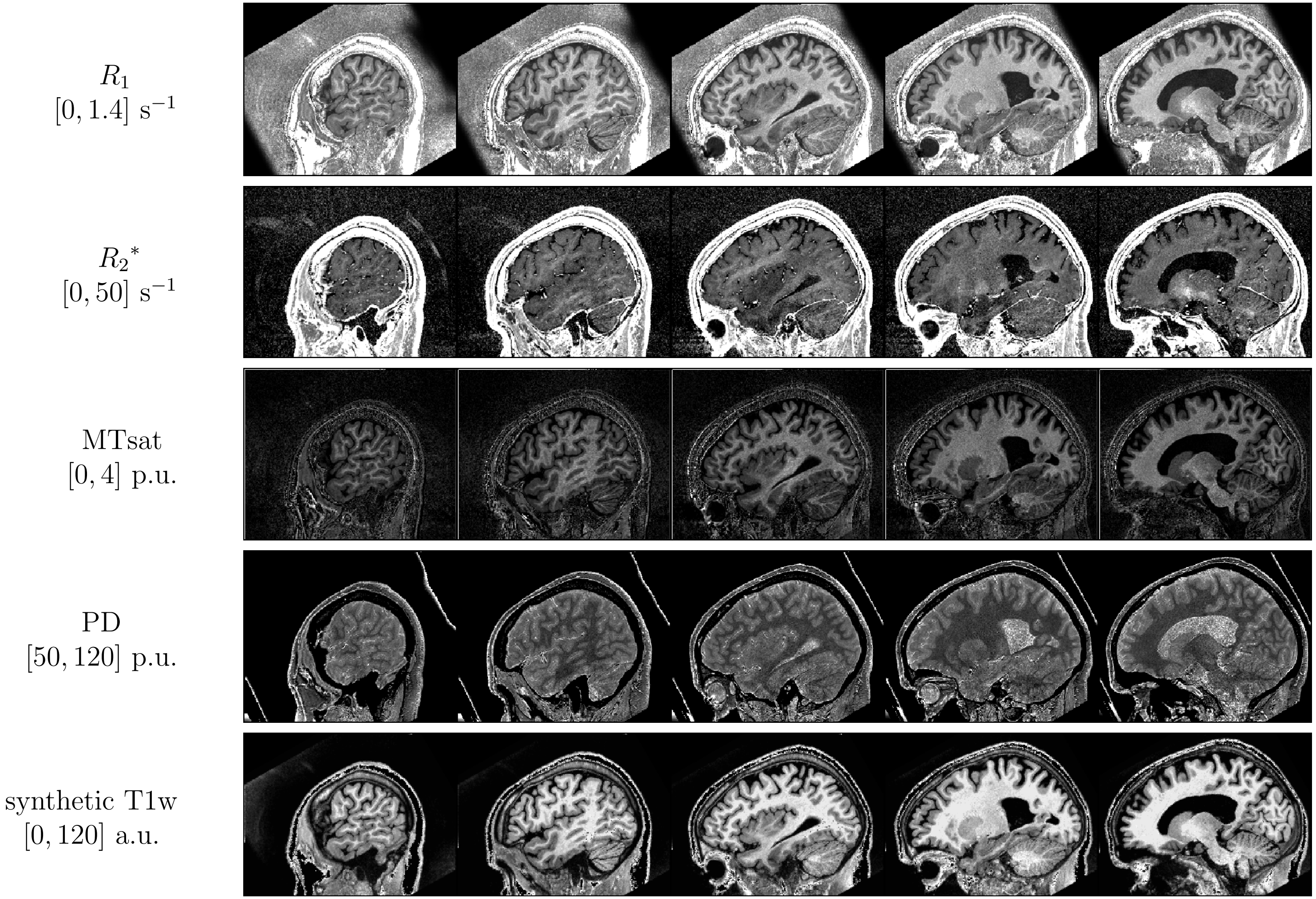

Figure S2: Sagittal slices of MPMs and synthetic images at 3T from the subject with the highest SD-$R_2{}^*$, i.e. the worst data quality. From left to right are slices of the left hemisphere going from lateral to medial. Only the left hemisphere is shown as only this hemisphere was investigated in this study. Even for this “worst” dataset the data quality is still good, though ringing artefacts are visible in the PDand $R_2{}^*$ maps which probably reflect some motion within the scans.

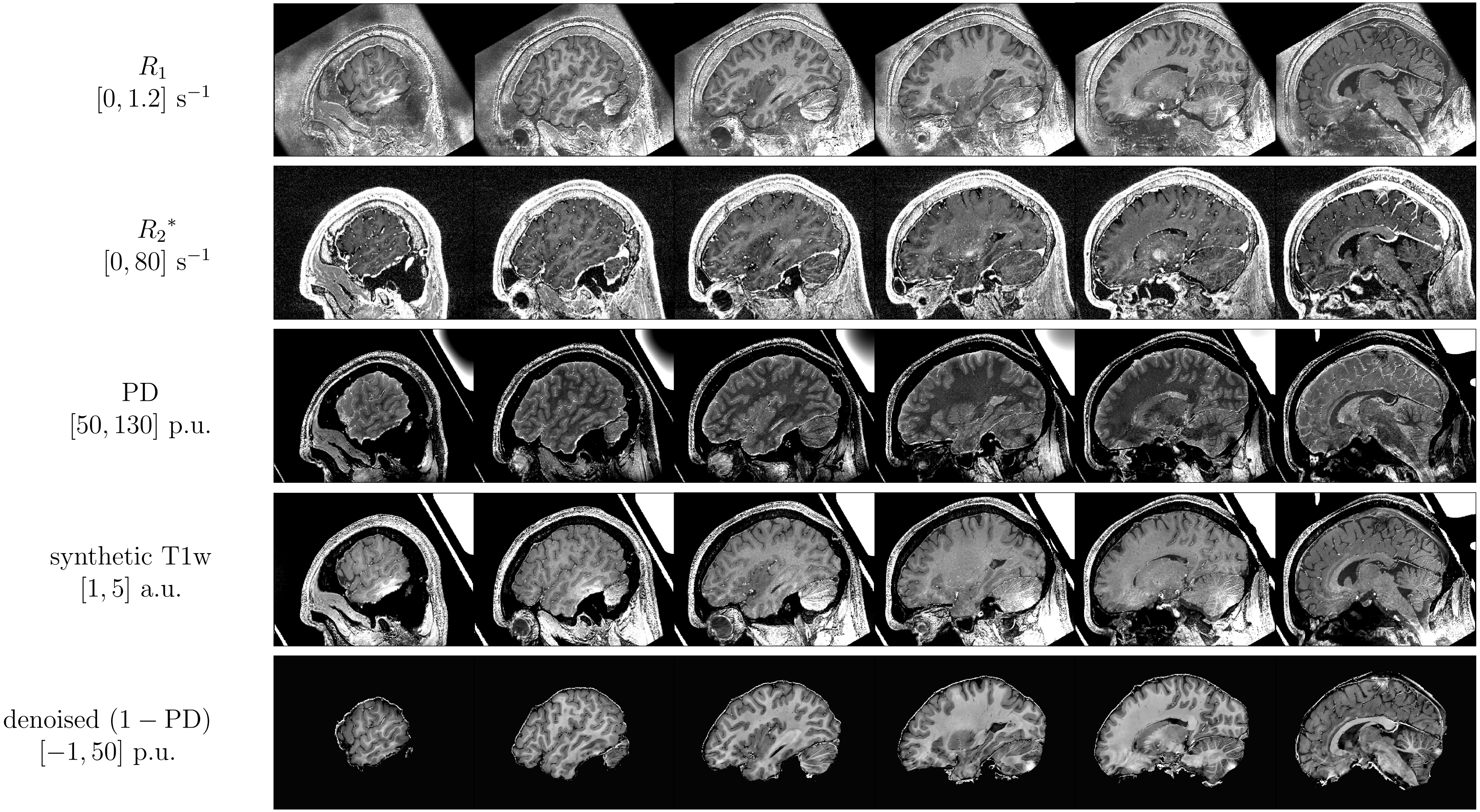

Figure S3: Sagittal slices of MPMs and synthetic images at 7T from the subject with the lowest SD-$R_2{}^*$, i.e. the best data quality. From left to right are slices of the left hemisphere going from lateral to medial. Only the left hemisphere is shown as only this hemisphere was investigated in this study. In the R_1 map, bright patchy artefacts can be seen in the inferior medial temporal lobe and inferior frontal lobe which likely result from low B_1 and high B_0 in these regions. These artefacts propagate to the synthetic T1w image.

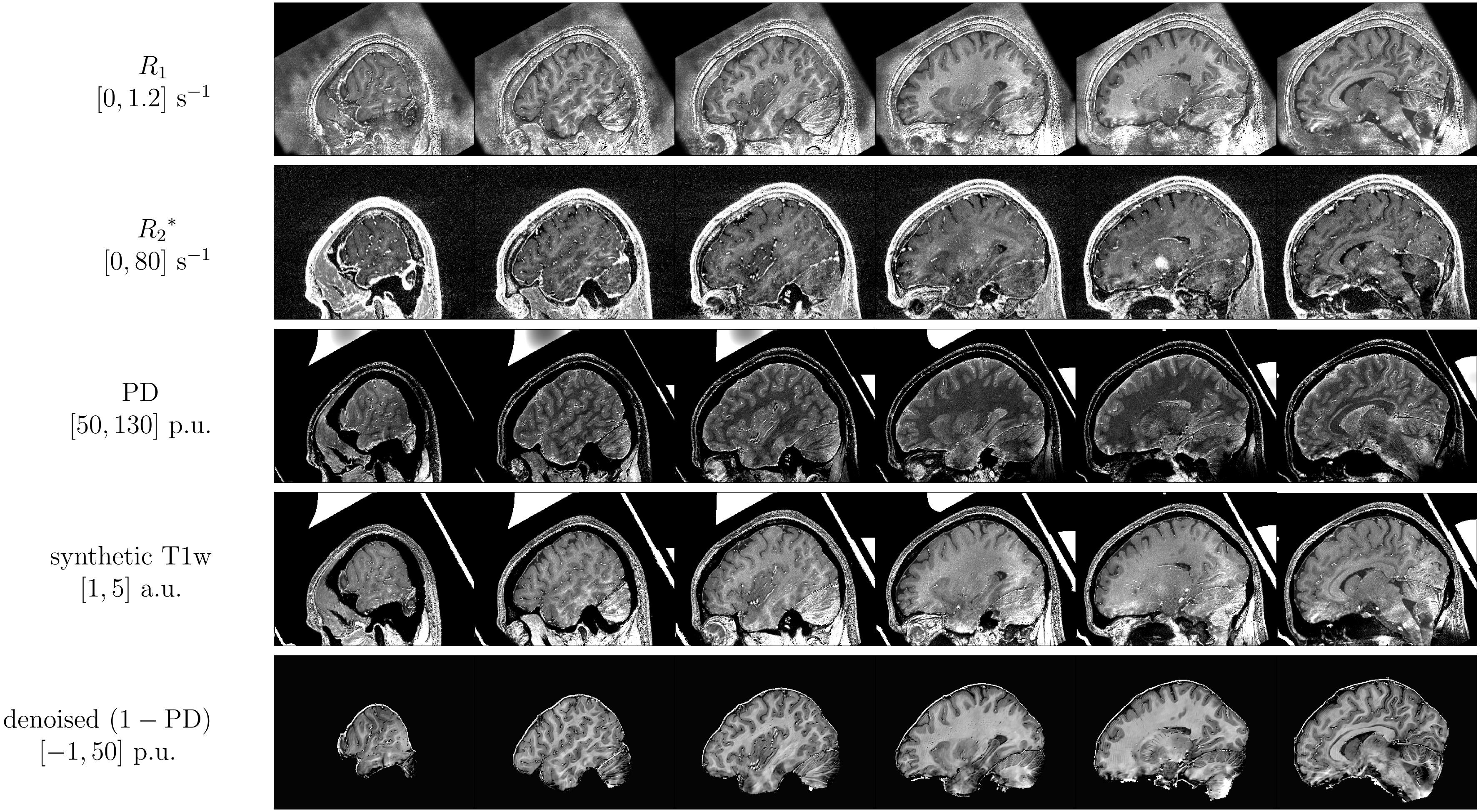

Figure S4: Sagittal slices of MPMs and synthetic images at 7T from the subject with the highest SD-$R_2{}^*$, i.e. the worst data quality. From left to right are slices of the left hemisphere going from lateral to medial. Only the left hemisphere is shown as only this hemisphere was investigated in this study. In the R_1 map, bright patchy artefacts can be seen in the inferior medial temporal lobe, the temporal pole, the inferior frontal lobe, and the white matter of the occipital lobe which likely result from low B_1 and high B_0 in these regions. These artefacts propagate to the synthetic T1w image. The data quality for this "worst" dataset is still comparable to the "best" 7T dataset (Figure S3). The location of the artefacts differs somewhat between this subject and the subject in Figure S3, implying that averaging over subjects will help to mitigate the impact of the artefacts on the R_1 results.

Flow chart showing gene expression analysis procedure

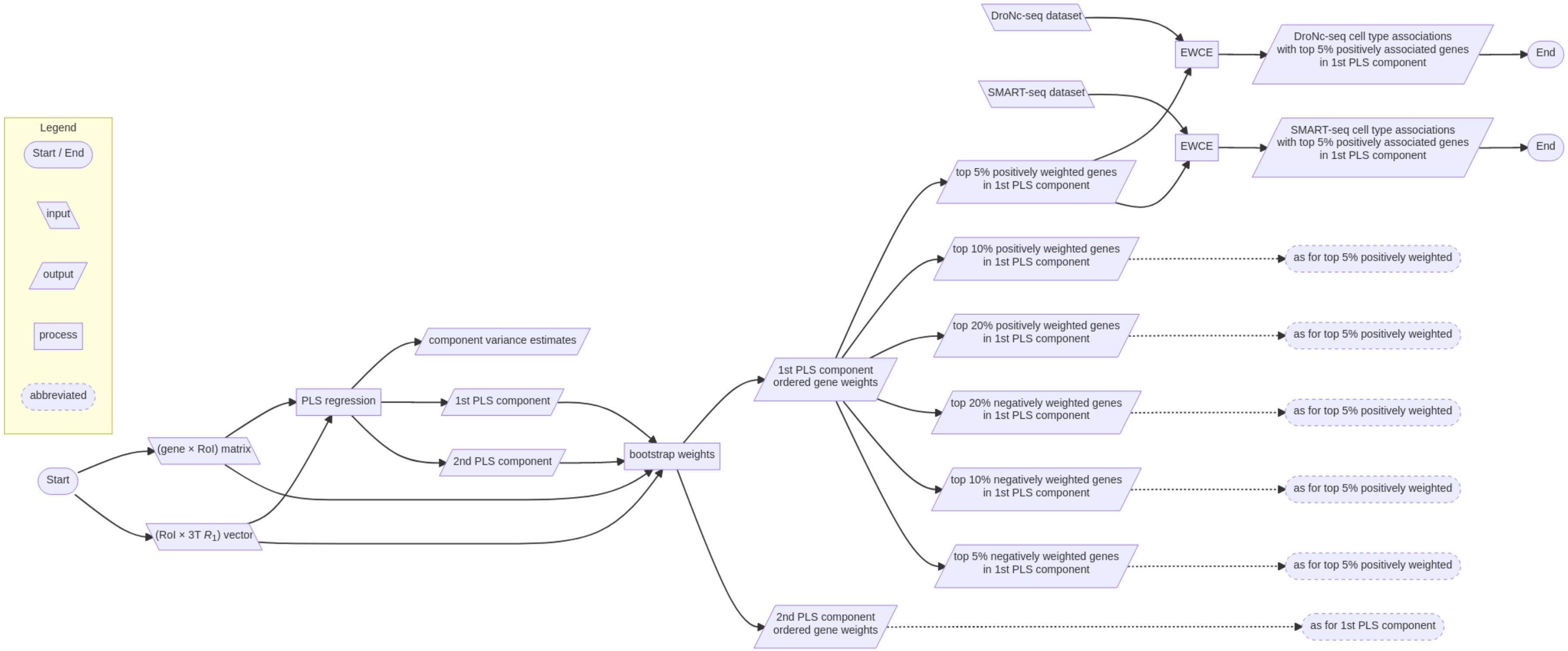

Figure S5: Flow chart showing how the (RoI $\times$ 3T R_1) vector is combined with gene expression data using PLS regression and EWCE to extract cell type associations. The terms and abbreviations used can be found in the Materials and Methods. R_1 at 3T is shown as an example, but the procedure is the same for each quantitative parameter and field strength.

Full EWCE results

Below are the complete results of the EWCE analyses. These results include cell types only present as a cell type in one of the cell type-specific datasets, which were for this reason omitted from the main manuscript as this meant a replication analysis could not performed (see Materials and Methods). They also include the results at the level of the top 10 and 20% of genes associated with each parameter in addition to those at the top 5% level (most of which were presented in Figures 3–5 in the main text).

Figures S6–S8 show the EWCE results with the SMART-seq dataset for the top 5, 10, and 20% of genes positively (upweighted) and negatively (downweighted) associated with each qMRI parameter in the first PLS component.

Figures S9–S11 show the EWCE results with the DroNc-seq dataset for the top 5, 10, and 20% of genes positively (upweighted) and negatively (downweighted) associated with each qMRI parameter in the first PLS component.

Figures S12–S14 show the EWCE results with the SMART-seq dataset for the top 5, 10, and 20% of genes positively (upweighted) and negatively (downweighted) associated with each qMRI parameter in the second PLS component.

Figures S15–S17 show the EWCE results with the DroNc-seq dataset for the top 5, 10, and 20% of genes positively (upweighted) and negatively (downweighted) associated with each qMRI parameter in the second PLS component.

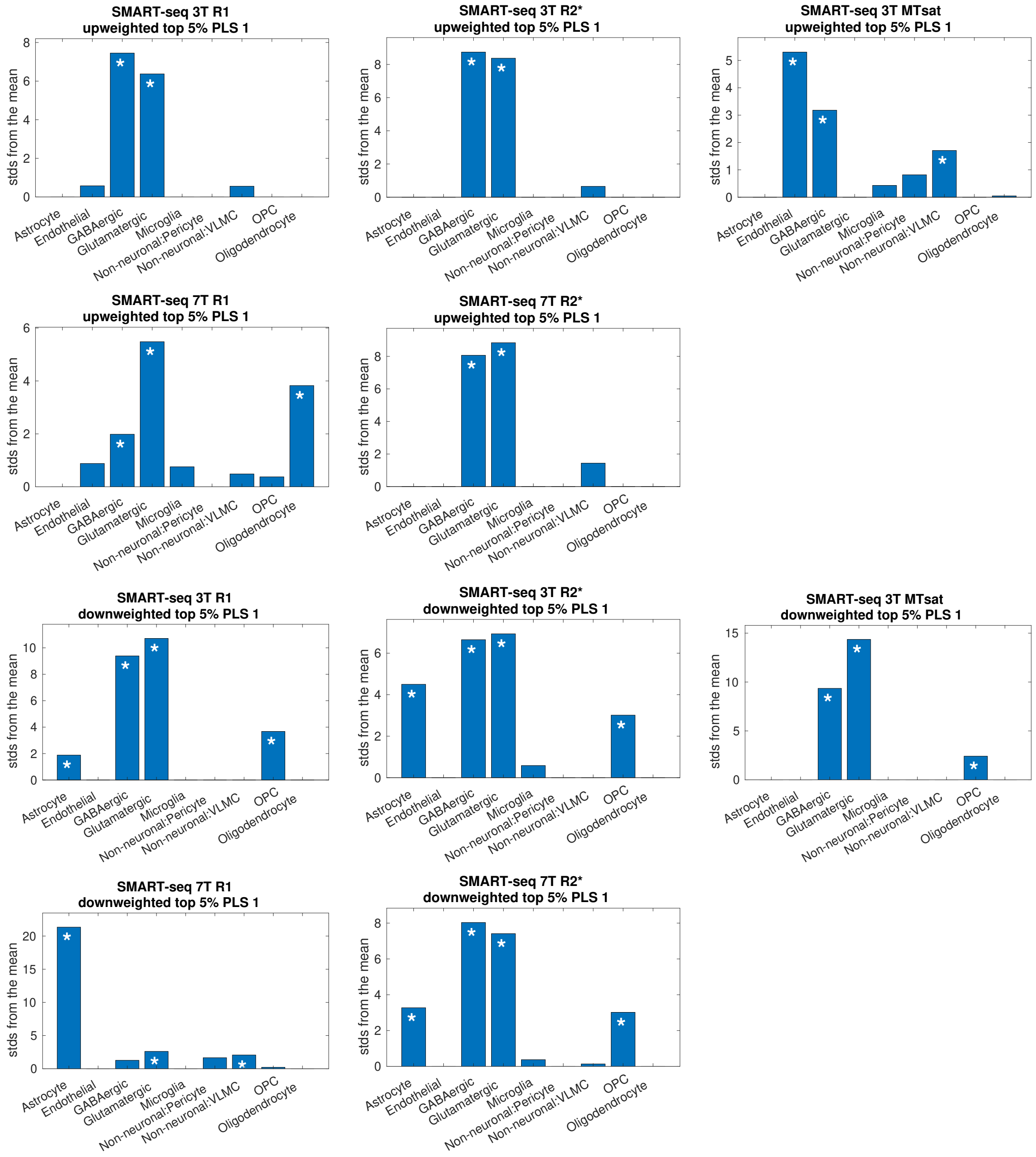

Figure S6: EWCE results for the top 5% of up- and downweighted genes associated with PLS component 1 of each parameter using SMART-seq gene lists. MT: MTsat, VLMC: vascular and leptomeningeal cells, OPC: oligodendrocyte precursor cells. Bars are only plotted when FDR-corrected $p < 0.5$. *: FDR-corrected $p < 0.05$.

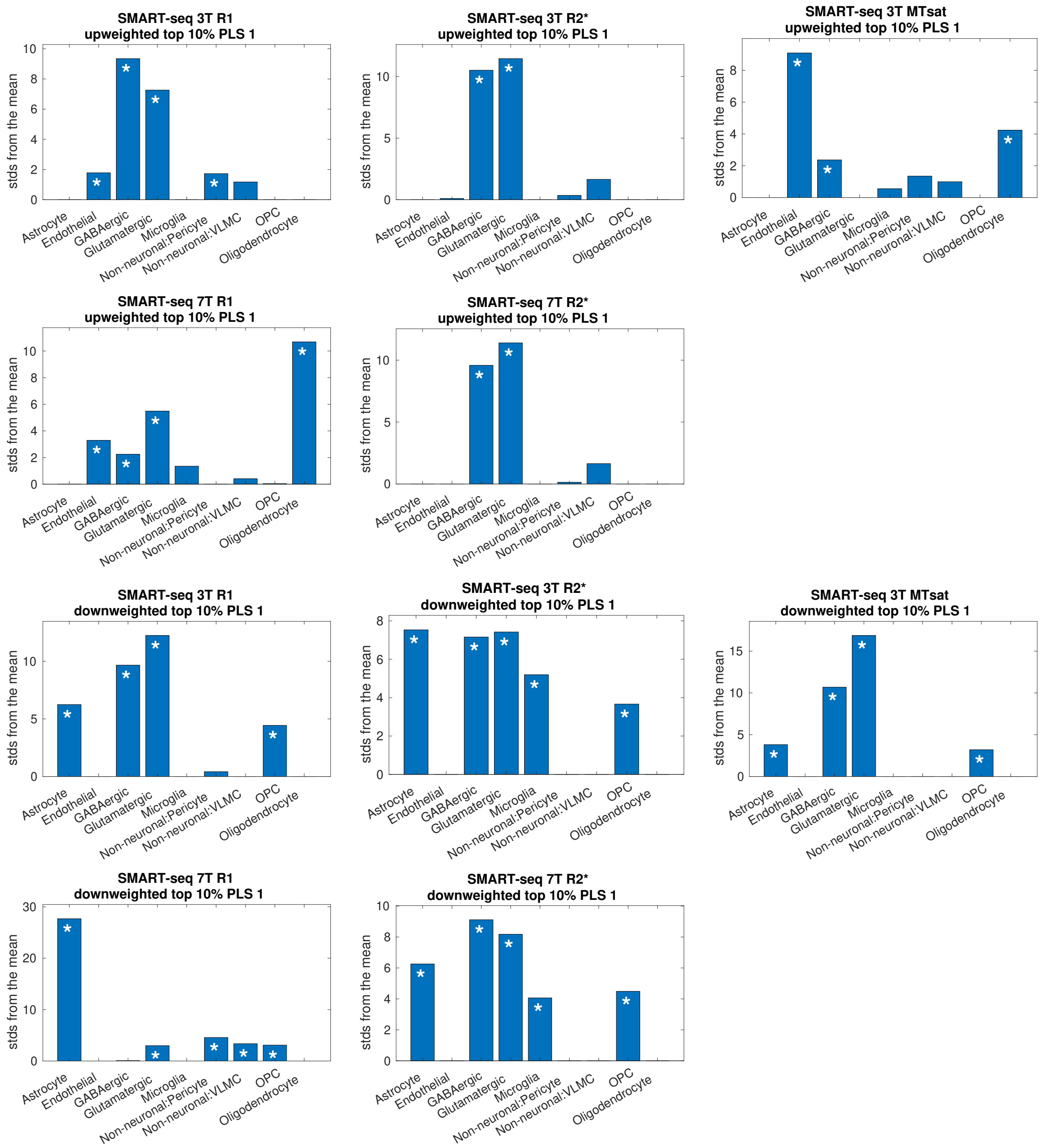

Figure S7: EWCE results for the top 10% of up- and downweighted genes associated with PLS component 1 of each parameter using SMART-seq gene lists. MT: MTsat, VLMC: vascular and leptomeningeal cells, OPC: oligodendrocyte precursor cells. Bars are only plotted when FDR-corrected $p < 0.5$. *: FDR-corrected $p < 0.05$.

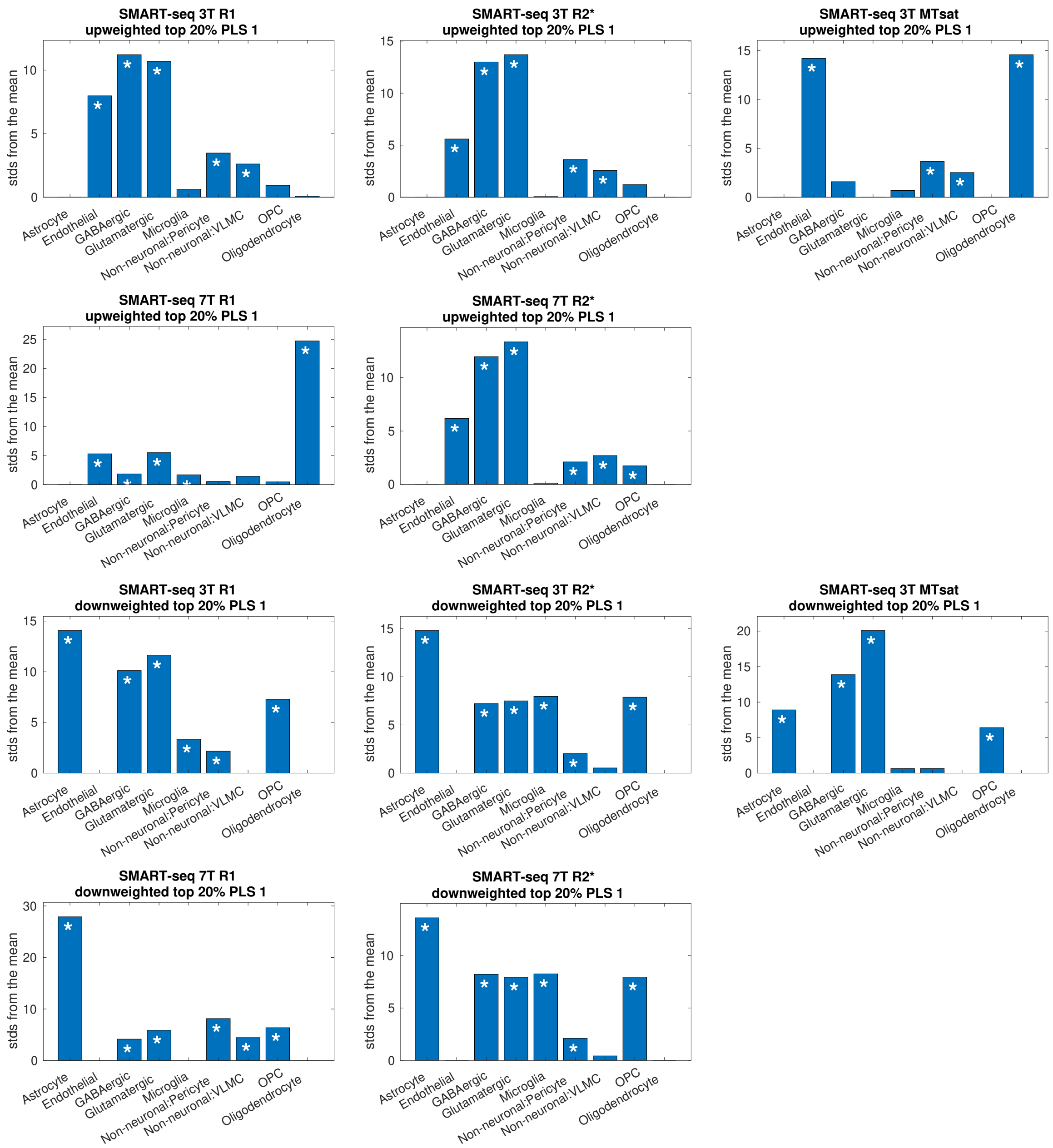

Figure S8: EWCE results for the top 20% of up- and downweighted genes associated with PLS component 1 of each parameter using SMART-seq gene lists. MT: MTsat, VLMC: vascular and leptomeningeal cells, OPC: oligodendrocyte precursor cells. Bars are only plotted when FDR-corrected $p < 0.5$. *: FDR-corrected $p < 0.05$.

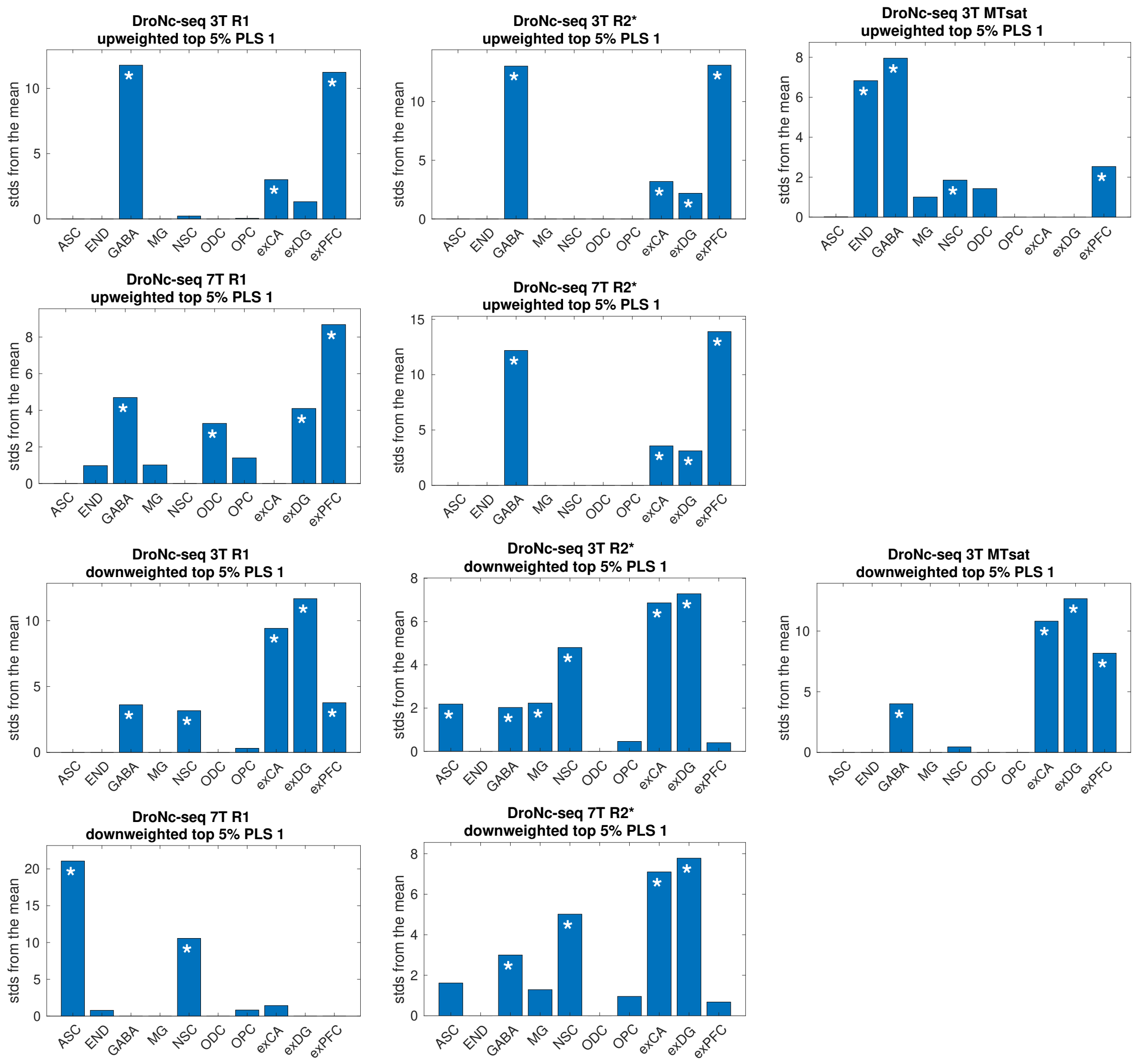

Figure S9: EWCE results for the top 5% of up- and downweighted genes associated with PLS component 1 of each parameter using DroNc-seq gene lists. MT: MTsat, ASC: astrocytes, END: endothelial cells, exCA: pyramidal neurons from the hippocampal CA region, exDG: granule neurons from the hippocampal dentate gyrus region, exPFC: glutamatergic neurons from the prefrontal cortex, GABA: GABAergic (inhibitory) neurons, MG: microglia, NSC: neuronal stem cells, ODC: oligodendrocytes, OPC: oligodendrocyte precursor cells. Bars are only plotted when FDR-corrected $p < 0.5$. *: FDR-corrected $p < 0.05$.

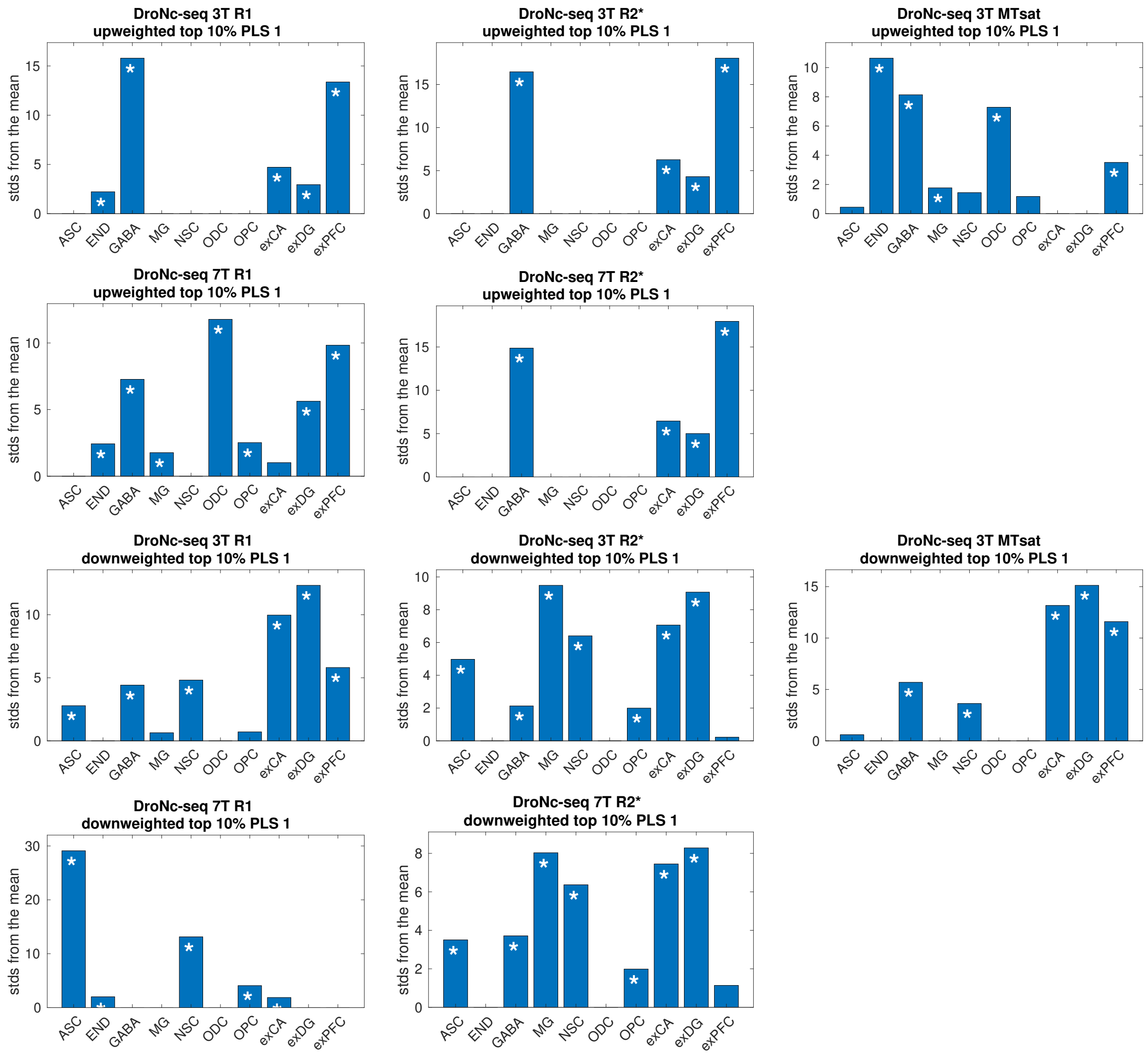

Figure S10: EWCE results for the top 10% of up- and downweighted genes associated with PLS component 1 of each parameter using DroNc-seq gene lists. MT: MTsat, ASC: astrocytes, END: endothelial cells, exCA: pyramidal neurons from the hippocampal CA region, exDG: granule neurons from the hippocampal dentate gyrus region, exPFC: glutamatergic neurons from the prefrontal cortex, GABA: GABAergic (inhibitory) neurons, MG: microglia, NSC: neuronal stem cells, ODC: oligodendrocytes, OPC: oligodendrocyte precursor cells. Bars are only plotted when FDR-corrected $p < 0.5$. *: FDR-corrected $p < 0.05$.

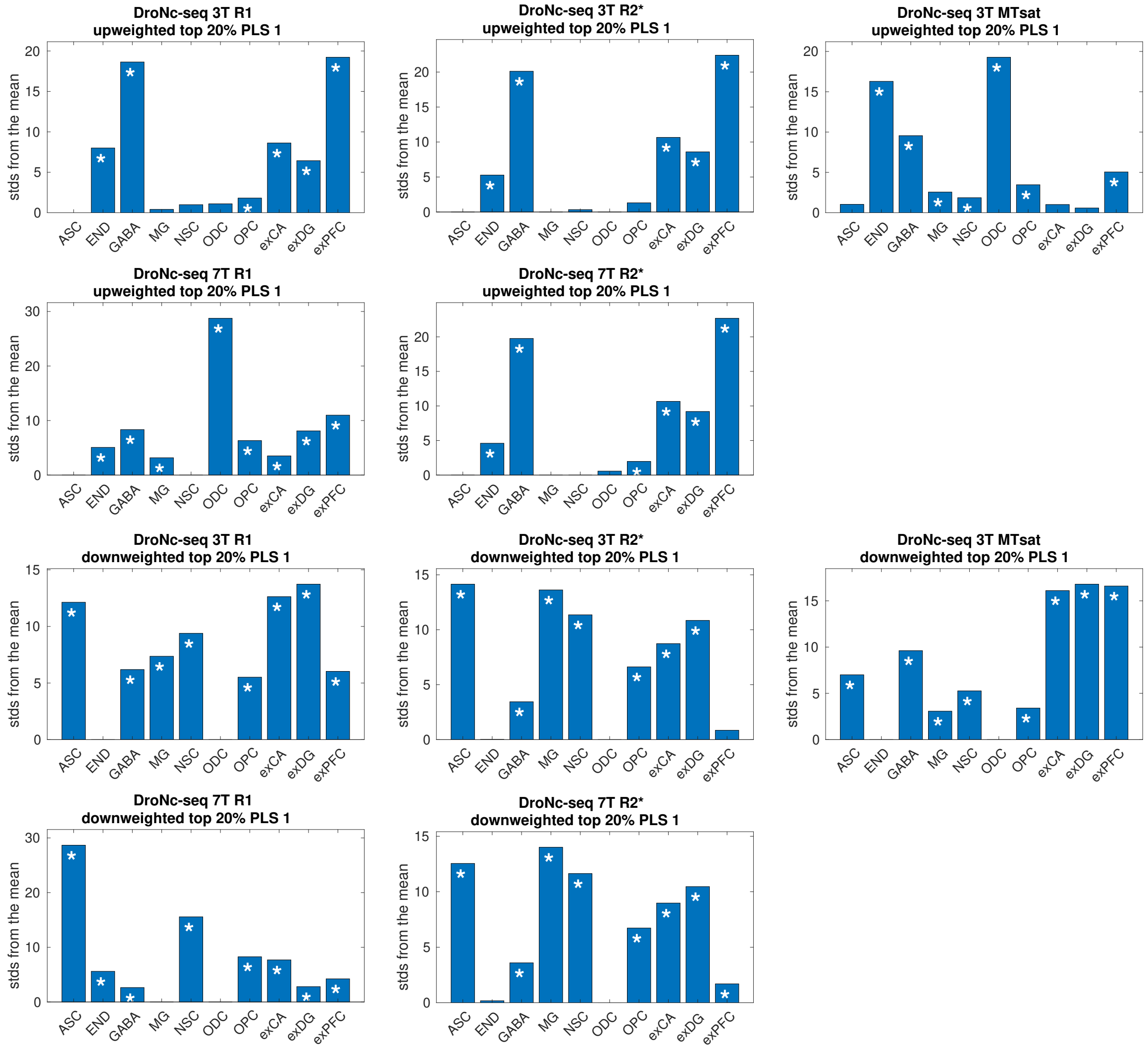

Figure S11: EWCE results for the top 20% of up- and downweighted genes associated with PLS component 1 of each parameter using DroNc-seq gene lists. MT: MTsat, ASC: astrocytes, END: endothelial cells, exCA: pyramidal neurons from the hippocampal CA region, exDG: granule neurons from the hippocampal dentate gyrus region, exPFC: glutamatergic neurons from the prefrontal cortex, GABA: GABAergic (inhibitory) neurons, MG: microglia, NSC: neuronal stem cells, ODC: oligodendrocytes, OPC: oligodendrocyte precursor cells. Bars are only plotted when FDR-corrected $p < 0.5$. *: FDR-corrected $p < 0.05$.

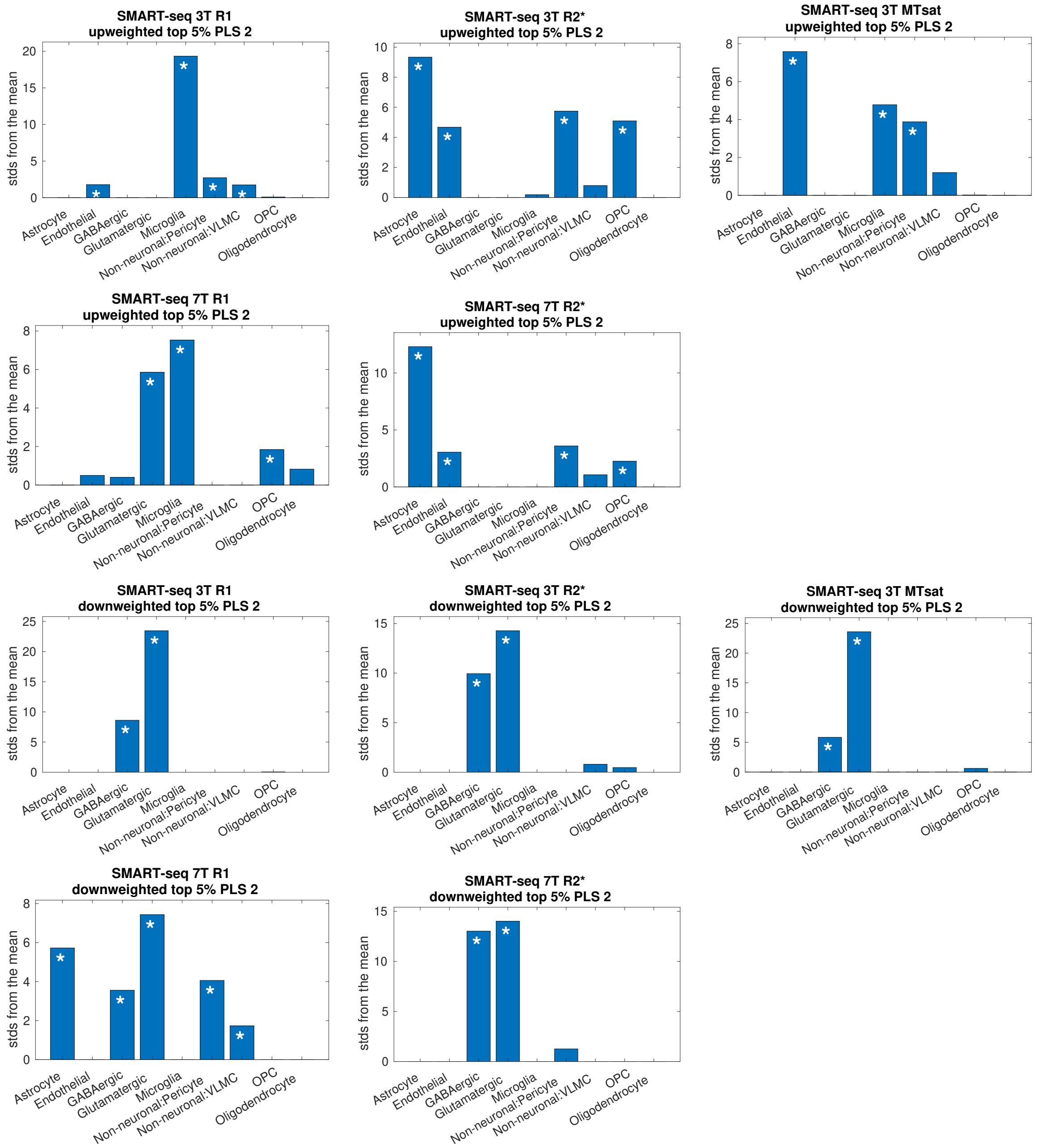

Figure S12: EWCE results for the top 5% of up- and downweighted genes associated with PLS component 2 of each parameter using SMART-seq gene lists. MT: MTsat, VLMC: vascular and leptomeningeal cells, OPC: oligodendrocyte precursor cells. Bars are only plotted when FDR-corrected $p < 0.5$. *: FDR-corrected $p < 0.05$.

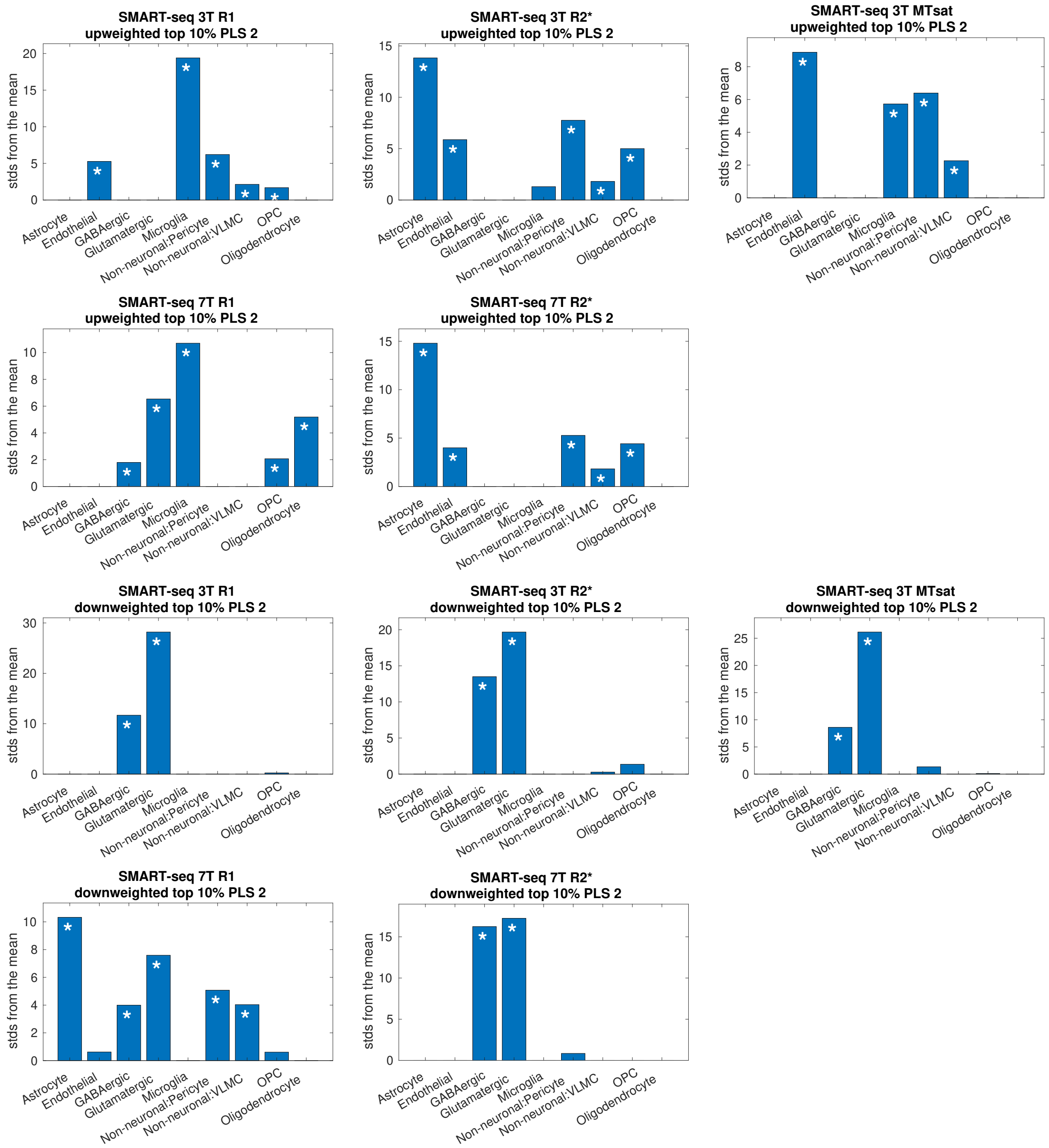

Figure S13: EWCE results for the top 10% of up- and downweighted genes associated with PLS component 2 of each parameter using SMART-seq gene lists. MT: MTsat, VLMC: vascular and leptomeningeal cells, OPC: oligodendrocyte precursor cells. Bars are only plotted when FDR-corrected $p < 0.5$. *: FDR-corrected $p < 0.05$.

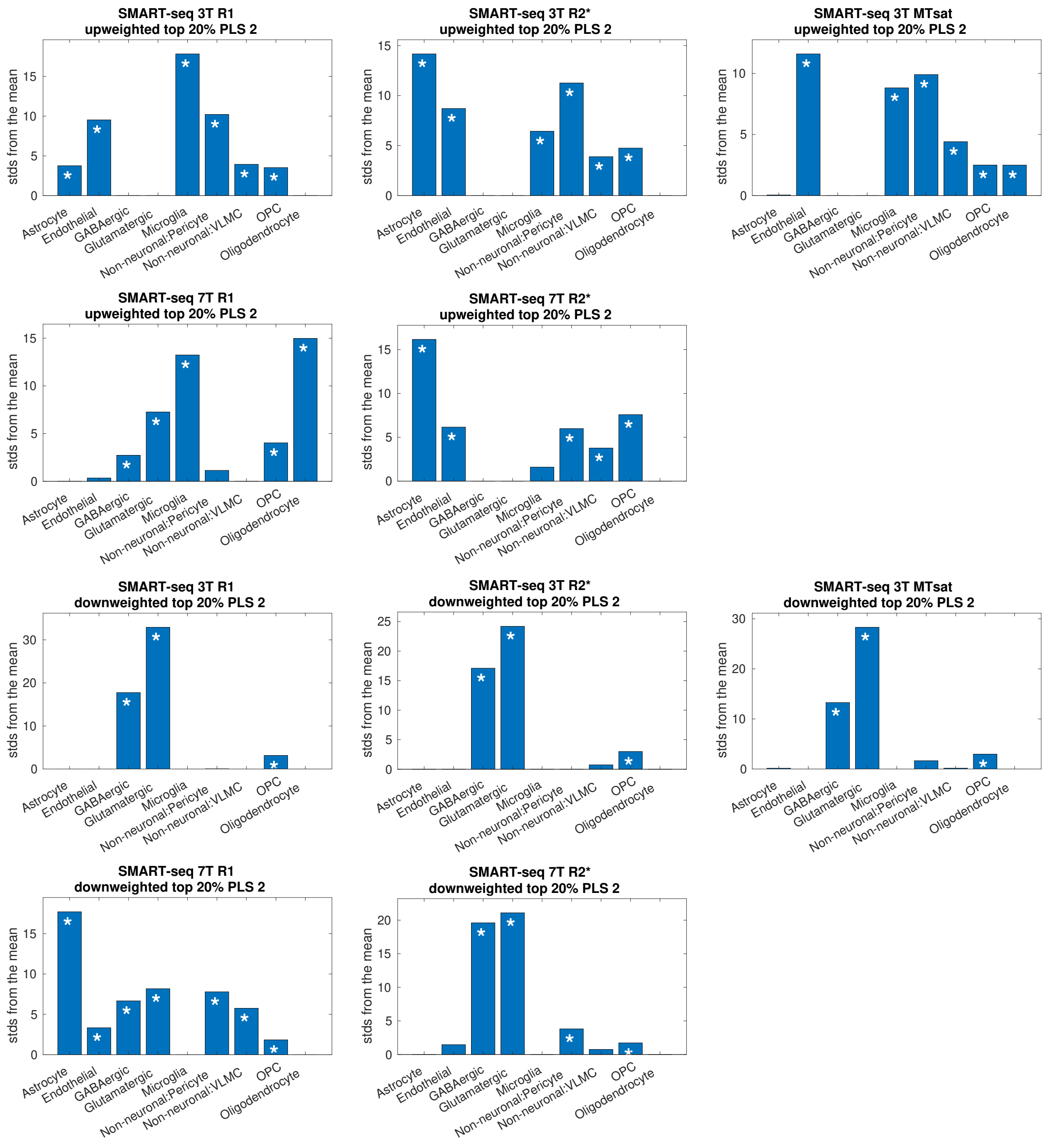

Figure S14: EWCE results for the top 20% of up- and downweighted genes associated with PLS component 2 of each parameter using SMART-seq gene lists. MT: MTsat, VLMC: vascular and leptomeningeal cells, OPC: oligodendrocyte precursor cells. Bars are only plotted when FDR-corrected $p < 0.5$. *: FDR-corrected $p < 0.05$.

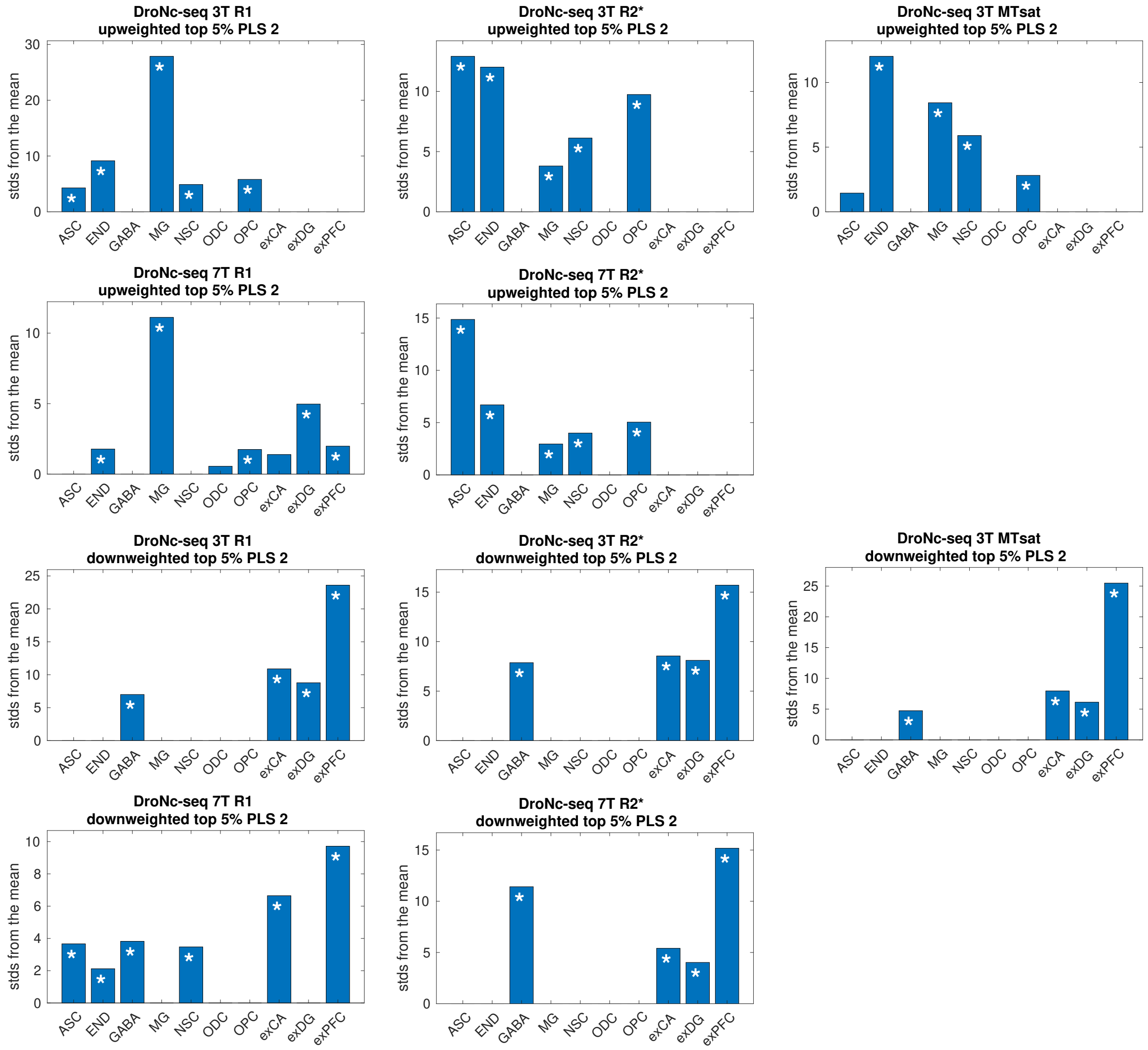

Figure S15: EWCE results for the top 5% of up- and downweighted genes associated with PLS component 2 of each parameter using DroNc-seq gene lists. MT: MTsat, ASC: astrocytes, END: endothelial cells, exCA: pyramidal neurons from the hippocampal CA region, exDG: granule neurons from the hippocampal dentate gyrus region, exPFC: glutamatergic neurons from the prefrontal cortex, GABA: GABAergic (inhibitory) neurons, MG: microglia, NSC: neuronal stem cells, ODC: oligodendrocytes, OPC: oligodendrocyte precursor cells. Bars are only plotted when FDR-corrected $p < 0.5$. *: FDR-corrected $p < 0.05$.

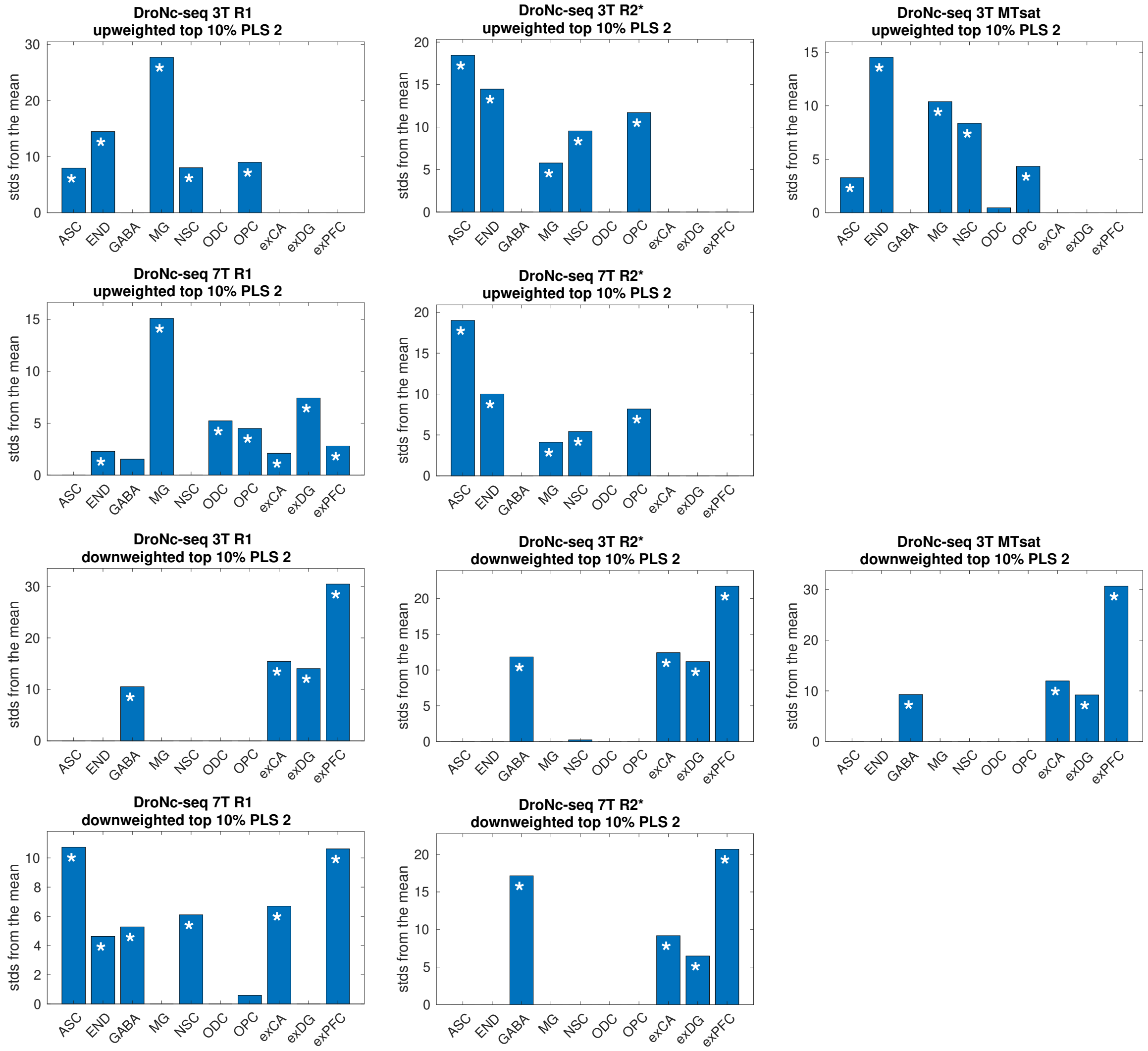

Figure S16: EWCE results for the top 10% of up- and downweighted genes associated with PLS component 2 of each parameter using DroNc-seq gene lists. MT: MTsat, ASC: astrocytes, END: endothelial cells, exCA: pyramidal neurons from the hippocampal CA region, exDG: granule neurons from the hippocampal dentate gyrus region, exPFC: glutamatergic neurons from the prefrontal cortex, GABA: GABAergic (inhibitory) neurons, MG: microglia, NSC: neuronal stem cells, ODC: oligodendrocytes, OPC: oligodendrocyte precursor cells. Bars are only plotted when FDR-corrected $p < 0.5$. *: FDR-corrected $p < 0.05$.

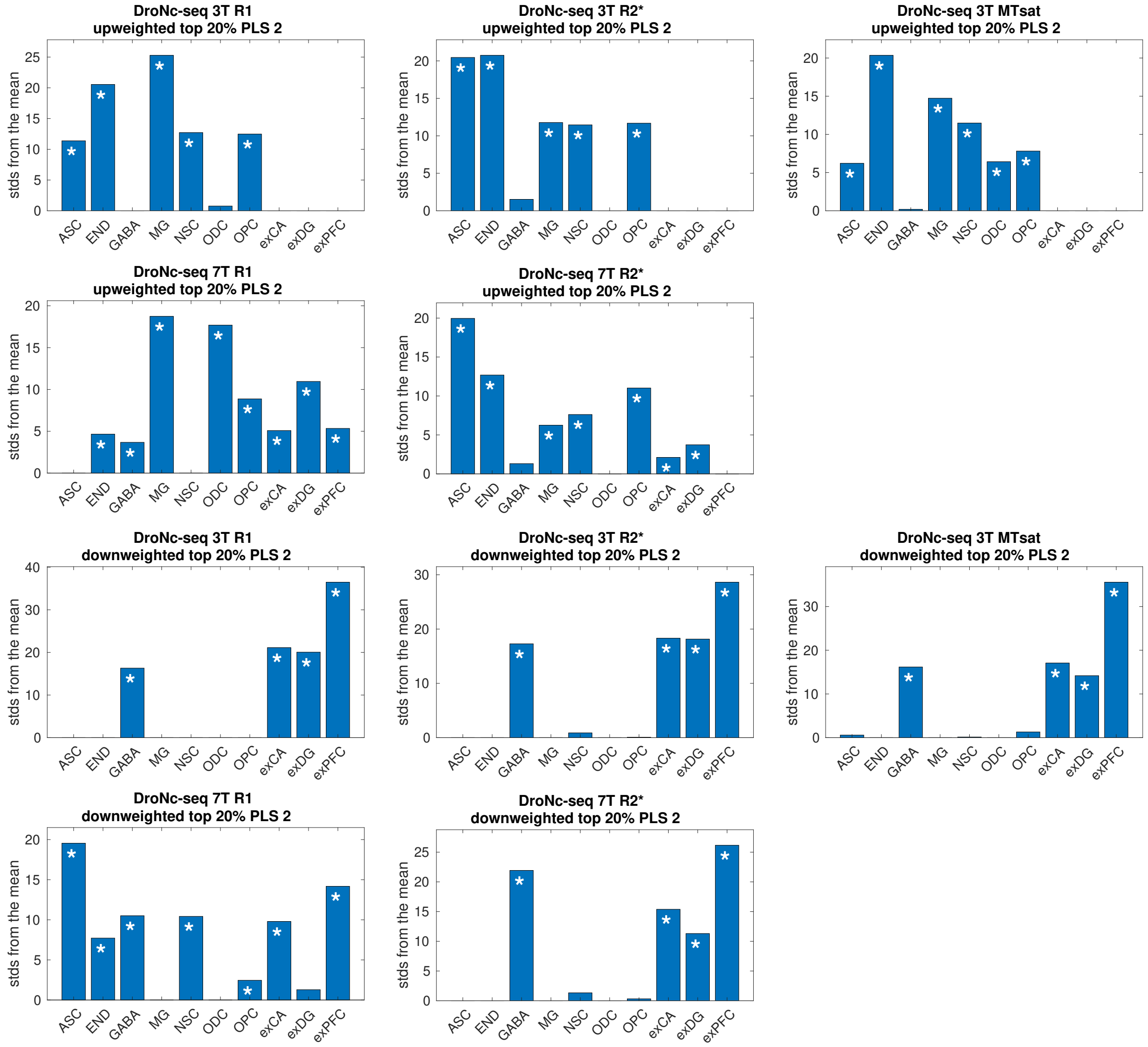

Figure S17: EWCE results for the top 20% of up- and downweighted genes associated with PLS component 2 of each parameter using DroNc-seq gene lists. MT: MTsat, ASC: astrocytes, END: endothelial cells, exCA: pyramidal neurons from the hippocampal CA region, exDG: granule neurons from the hippocampal dentate gyrus region, exPFC: glutamatergic neurons from the prefrontal cortex, GABA: GABAergic (inhibitory) neurons, MG: microglia, NSC: neuronal stem cells, ODC: oligodendrocytes, OPC: oligodendrocyte precursor cells. Bars are only plotted when FDR-corrected $p < 0.5$. *: FDR-corrected $p < 0.05$.

R_1 7T results including potentially artefact-affected areas

The locations of the areas which were omitted for the R_1 7T analysis in the main text are shown in Figure S18 projected on the Freesurfer `fsaverage` brain. Comparison with Figures S3 and S4 shows that they correspond well with the bulk of the bright patchy cortical artefacts in the 7T R_1 maps.

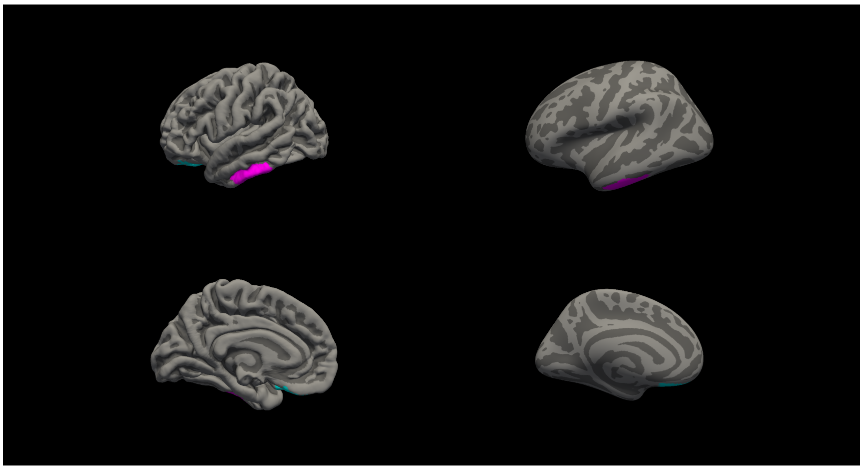

Figure S18: The areas in the HCP-MMP1.0 atlas (Glasser et al., 2016) potentially affected by artefacts in the 7T R_1 maps plotted on the left hemisphere of the `fsaverage` brain. Cyan: orbitofrontal complex; magenta: area TE2 anterior. Left: areas plotted on the pial surface for comparison with Figures S1–S4. Right: areas plotted on the inflated surface for comparison with Figure 1. Top: lateral view. Bottom: medial view.

Below are the results of the EWCE analyses for both the SMART-seq and DroNc-seq cell type-specific datasets with gene lists comprising the top 5, 10, and 20% of genes positively (upweighted) and negatively (downweighted) associated with R_1 at 7T in each PLS component when including areas potentially strongly affected by B_1 and B_0 artefacts (see Materials and Methods). Table S1 shows the variance explained in the PLS components when including these areas, Figure S19 shows the results for the top 5% of genes associated with R_1 at 7T (cf. Figure 5), and Figures S20 and S21 show the full EWCE results for the first and second PLS component, respectively (cf. Figures S6–S17).

	PLS component	Spatial variance explained in: gene distribution	qMRI parameter
R_1 7T	1	12%	22%
	2	15%	12%

Table S1: Variance explained by the PLS components for R_1 at 7T when including areas potentially strongly affected by B_1 and B_0 artefacts (cf. Table 1 in the main manuscript).

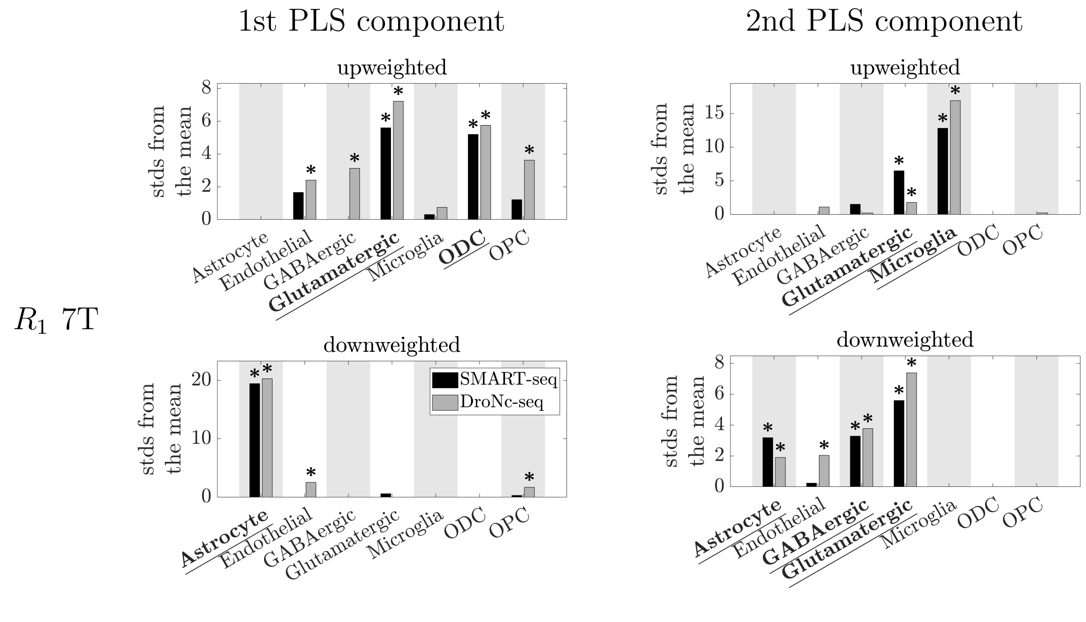

Figure S19: EWCE results showing the cell type associations of the top 5% of genes associated with R_1 at 7T when including inferior regions associated with B_1 and B_0 artefacts (cf. Figure 5 in the main manuscript). Plotted are the number of standard deviations (stds) by which the EWCE value deviated from the mean value over bootstrapped target lists. Results from the two cell type-specific datasets are plotted in different colours: SMART-seq in black, DroNc-seq in grey. Left: First component of the PLS. Right: Second component of the PLS. Bars are only plotted when FDR-corrected $p < 0.5$. *: FDR-corrected $p < 0.05$. Significant cell type associations which replicated between both cell type-specific datasets (robust results) are underlined and in bold.

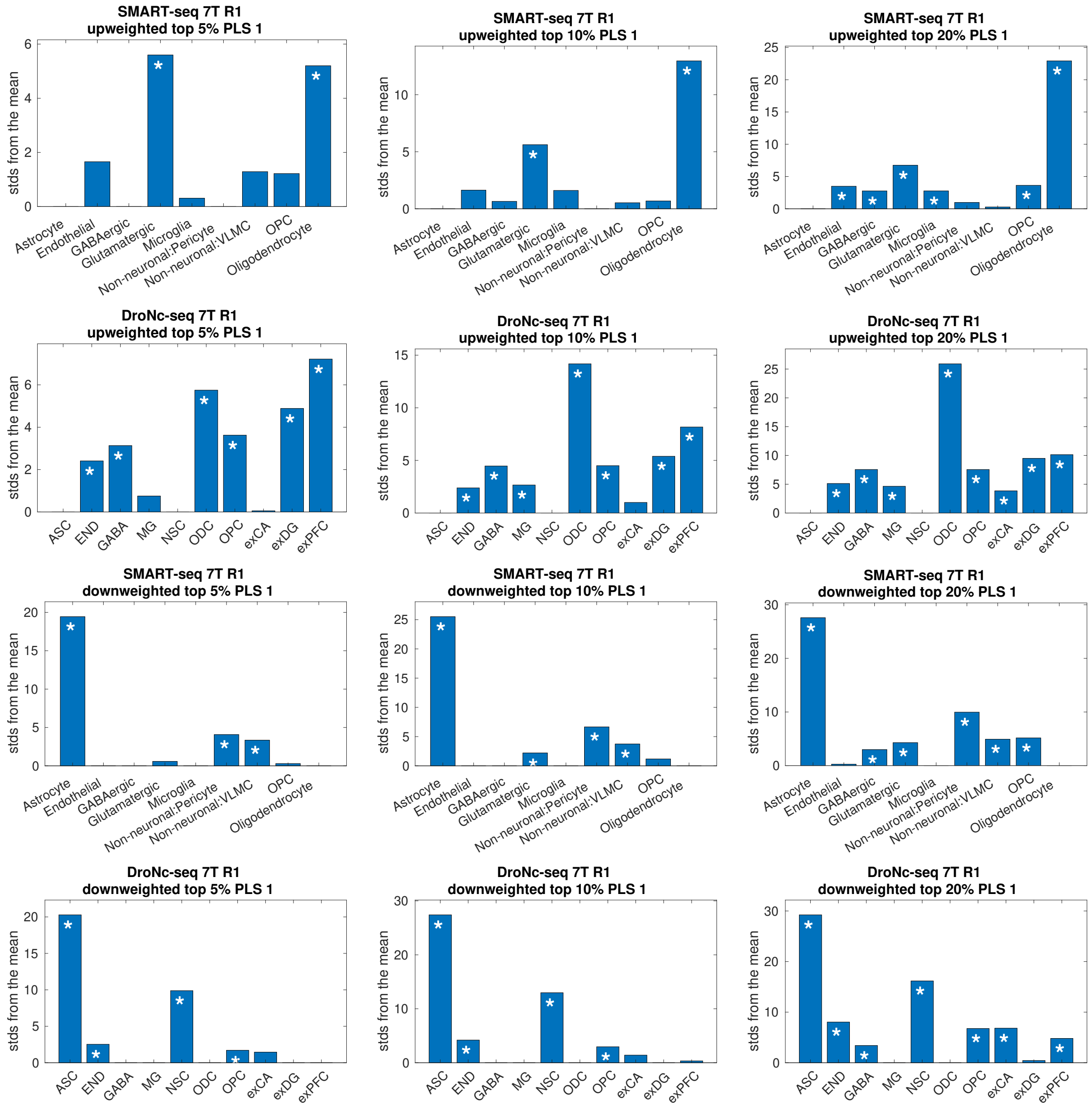

Figure S20: EWCE results on top 5, 10 and 20% of up- and downweighted genes associated with PLS component 1 of R_1 in both cell type-specific gene lists when including inferior regions associated with B_1 and B_0 artefacts. ASC: astrocytes, END: endothelial cells, exCA: pyramidal neurons from the hippocampal CA region, exDG: granule neurons from the hippocampal dentate gyrus region, exPFC: glutamatergic neurons from the prefrontal cortex, GABA: GABAergic (inhibitory) neurons, MG: microglia, NSC: neuronal stem cells, ODC: oligodendrocytes, OPC: oligodendrocyte precursor cells, VLMC: vascular and leptomeningeal cells. Bars are only plotted when FDR-corrected $p < 0.5$. *: FDR-corrected $p < 0.05$.

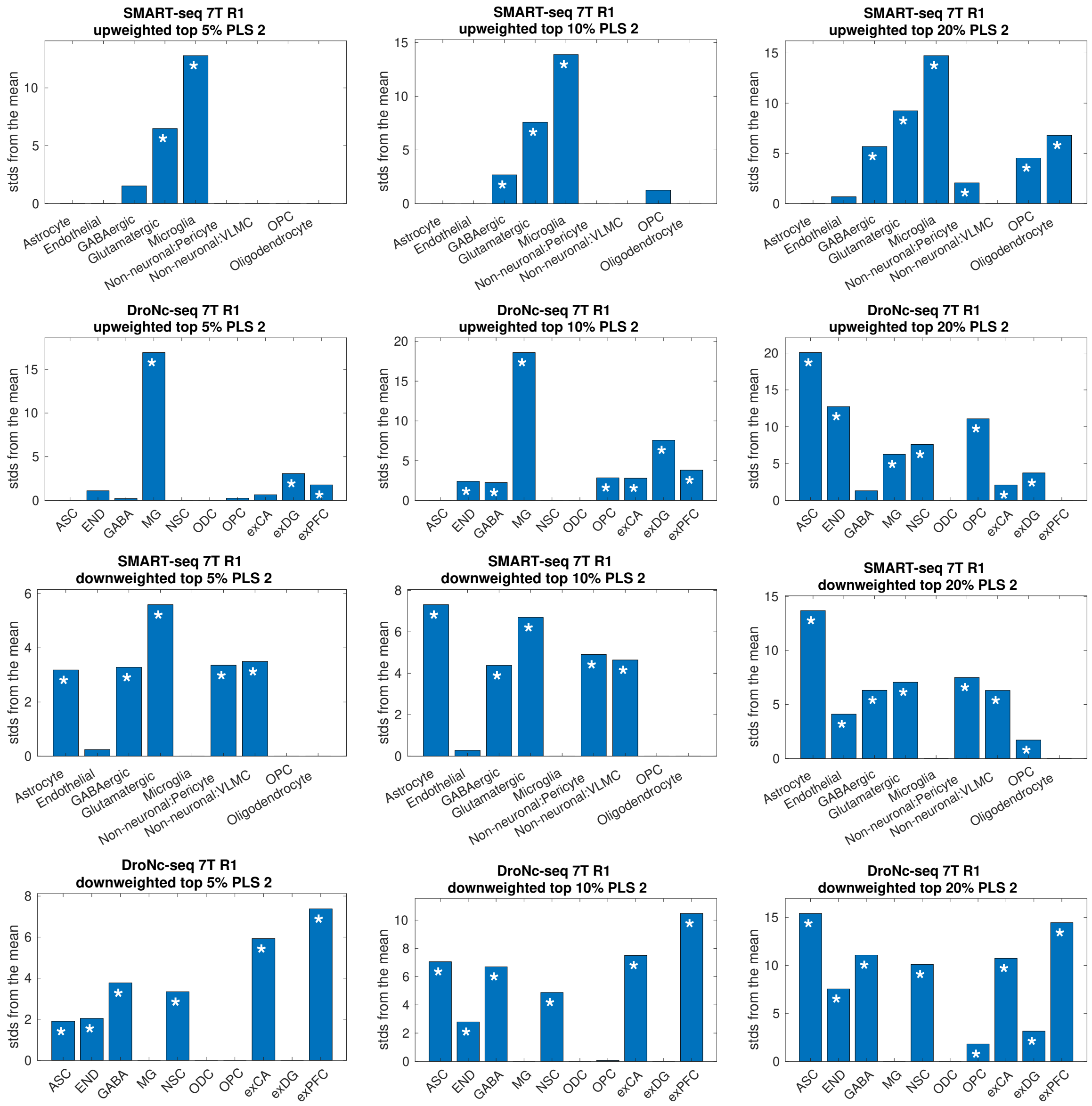

Figure S21: EWCE results on top 5, 10 and 20% of up- and downweighted genes associated with PLS component 2 of R_1 in both cell type-specific gene lists when including inferior regions associated with B_1 and B_0 artefacts. ASC: astrocytes, END: endothelial cells, exCA: pyramidal neurons from the hippocampal CA region, exDG: granule neurons from the hippocampal dentate gyrus region, exPFC: glutamatergic neurons from the prefrontal cortex, GABA: GABAergic (inhibitory) neurons, MG: microglia, NSC: neuronal stem cells, ODC: oligodendrocytes, OPC: oligodendrocyte precursor cells, VLMC: vascular and leptomeningeal cells. Bars are only plotted when FDR-corrected $p < 0.5$. *: FDR-corrected $p < 0.05$.
